# Household‐level consumption data can be redistributed for individual‐level Optifood diet modeling: analysis from four countries

**DOI:** 10.1111/nyas.14709

**Published:** 2021-11-30

**Authors:** Frances Knight, Monica Woldt, Kavita Sethuraman, Gilles Bergeron, Elaine Ferguson

**Affiliations:** ^1^ Department of Population Health London School of Hygiene and Tropical Medicine London UK; ^2^ United Nations World Food Programme Rome Italy; ^3^ Helen Keller International Washington District of Columbia; ^4^ Formerly with the Food and Nutrition Technical Assistance Project (FANTA) Washington District of Columbia; ^5^ USAID Advancing Nutrition Arlington Virginia; ^6^ New York Academy of Sciences New York New York

**Keywords:** linear programming, household consumption and expenditure surveys, infant and young child feeding, food‐based recommendations

## Abstract

A barrier to using Optifood linear programming (LP), which identifies nutrient gaps and supports population‐specific food‐based recommendation (FBR) development, is the requirement for dietary intake data. We investigated whether Household Consumption and Expenditure Surveys (HCESs) could be used instead of individual‐level 24‐h recalls (24HRs). The 24HR data from 12‐ to 23‐month‐old breastfeeding children in rural Kenya, Uganda, Guatemala, and Bangladesh were paired with HCES food consumption data from similar areas (*n* = 8) and time periods. HCES food intakes (g/week) were estimated using adult male equivalents, adjusted for breastfeeding. Paired HCES‐ and 24HR‐defined LP inputs and outputs were compared using percentage agreement. Mean overall percentage agreements were 42%, 63%, and 80%, for food, food subgroup, and food‐group model parameters, respectively. HCES food lists were on average 1.3 times longer than 24HR. Similar nutrient gaps (77–100% agreement), food sources of nutrients (71–100% agreement), and FBRs (80–100% agreement) were identified. The results suggest that HCES data can be used in Optifood analyses for 12‐ to 23‐month‐old children, despite recognized challenges of using it to estimate dietary intakes of young children compared with older age groups. Further analyses, however, are required for different age groups and locations to confirm expectations that it would perform equally well.

## Graphical abstract

A barrier to using Optifood linear programming, which identifies nutrient gaps and supports population‐specific food‐based recommendation development, is the requirement for dietary intake data. We investigated whether Household Consumption and Expenditure Surveys (HCES) could be used instead of individual‐level 24‐h recalls (24HRs). The 24HR data from 12–23‐month‐old breastfeeding children in rural Kenya, Uganda, Guatemala, and Bangladesh were paired with HCES food consumption data from similar areas and time periods.

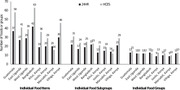

## Introduction

Linear programming (LP) has been used in tools based on mathematical modeling, such as the Optifood software, to identify “problem nutrients” (nutrients for which requirements would be difficult to meet using local foods within existing dietary patterns) and the best food sources of nutrients in the local food supply to provide them and to develop evidence‐based, population‐specific food‐based recommendations (FBRs).^1^, ^2^, ^3^, ^4^, ^5^ LP can also be used to estimate the nutritional benefits of providing access to nutritious agricultural products, supplements, fortified foods, or special recipes, in the context of the local diet,^3^, ^6^, ^7^, ^8^ and has been applied to inform nutrition and agricultural program decisions in low‐ and middle‐income countries (LMICs).^9^, ^10^, ^11^, ^12^ LP, therefore, is a robust modeling approach to inform both nutrition‐specific and ‐sensitive programming.

Optifood contains four analytical modules. Module 1 tests model parameters to ensure realistic diets will be generated. Module 2 identifies the nutritionally best diets within and outside of the population's average food consumption patterns to identify problem nutrients and best local food sources of nutrients that are used to decide on FBRs to test. Module 3 then tests and compares alternative sets of FBRs in terms of the level of nutrient adequacy that can be met if they are put into practice within the context of current diets. The optional fourth module optimizes diets based on cost.^13^

A key factor limiting the wider application of Optifood LP analysis to inform nutrition programs has been the paucity of quantitative dietary intake data to define the model parameters. Modeling realistic diets using LP analysis in Optifood requires data on the current food consumption patterns of a specific population (defined by age, sex, and region), including food types, quantities consumed, and frequency of consumption.^13^ Individual 24‐h recall (24HR) data are the most common source used to generate Optifood LP input parameters.^1^, ^2^, ^3^, ^5^, ^14^, ^15^, ^16^, ^17^ Unfortunately, the collection of quantitative individual‐level dietary intake data, through 7‐day observed‐weighed food records (the gold standard) or 24HRs, is complex and expensive in terms of time and resources.^18^, ^19^, ^20^ These data constraints often make LP analyses unfeasible,^18^, ^21^, ^22^, ^23^ which severely limits opportunities for evidence‐based programmatic decision making using LP.^22^

One potential source of routinely collected food consumption data for LP analyses is nationally representative and publicly accessible Household Consumption and Expenditure Surveys (HCESs). These surveys include Household Budget Surveys, Living Standards Measurement Surveys, Household Income and Expenditure Surveys, and Integrated Household Surveys.^18^, ^24^ These multipurpose surveys are conducted in over 120 countries to measure poverty and consumption patterns for consumer price index calculations^25^ and collect, among other indicators, data on household food consumption and availability.^26^ HCESs are being conducted with greater regularity, rigor, and quality, and datasets are becoming more available to the public.^26^, ^27^, ^28^

Many HCESs include food consumption modules that use closed food questionnaires to ask respondents which foods were consumed by household members over the past 7 or 14 days and their quantities. Increasingly, while not designed for this purpose, household‐level consumption data are being repurposed to estimate individual‐level food consumption.^25^, ^28^ The most common method, for redistributing food consumed at the household‐level to individuals within the same household, is the use of adult male consumption equivalent (AME). In this approach, the estimated energy requirement of each family member is divided by those of an average adult male,^26^, ^29^ and the AMEs of all household members are summed to give a total household AME. Each quantity of food reported as being consumed within a household is then redistributed among individuals within that household according to their AME quotient, whereby an individual's AME is divided by the household AME and multiplied by the quantity of each food.^29^

Validation studies comparing AME‐redistributed HCES food consumption data with those derived from 24HRs show that estimates of population‐level food and nutrient intakes, nutrient intake gaps, and/or prevalence of inadequacies in Bangladesh, Uganda, Ethiopia, Cameroon, and Mongolia are similar.^30^, ^31^, ^32^, ^33^, ^34^, ^35^, ^36^ However, three recent studies caution against using these methods for estimating dietary intakes of breastfeeding infants and young children, whose dietary patterns may differ from older age groups, because the methods tend to overestimate energy and nutrient intakes in this age group.^30^, ^34^, ^35^ However, this limitation could be addressed by assuming that breastmilk provides on average the estimated 67%, 55%, and 39% of the median energy requirements for infants aged 6–8, 9–11, and 12–23 months, respectively.^37^ To our knowledge, the use of HCES data for estimating LP model parameters for dietary modeling has not been validated. Its use for estimating LP model parameters for children under 2 years of age would expand opportunities to design evidence‐based national‐ or regional‐level complementary feeding recommendations globally.

Given the potential of using HCES data as an LP input when quantitative dietary data are not available, in this study, we aimed to determine the percentage agreement between LP input parameters generated using AME‐redistributed HCES consumption data and individual 24‐HR data. We used the LP input parameters from the HCES data and the 24‐HR data separately in the Optifood LP software to identify “problem nutrients” and formulate FBRs for 12‐ to 23‐month‐old children. We then compared the problem nutrients and FBRs generated using the two sets of data. We conducted this analysis in LMICs located in eight diverse geographical regions. The results of this study will indicate whether HCES data can be used to develop model parameters and generate FBRs using LP analyses in Optifood. These FBRs could then be field tested and used to inform programmatic decisions for infant and young child nutrition.

## Materials and methods

### Data sources

Pairs of individual‐ and household‐level food consumption data were identified. The inclusion criteria for these datasets were that they were (1) collected from 12‐ to 23‐month‐old children living in an LMIC, (2) the sample size of each 24HR dataset was ≥50 children, (3) paired HCES‐derived household‐consumption data and individual‐level dietary data were available for the same geographic area, (4) the paired HCES and 24HR datasets were collected within approximately 3 years of each other, and (5) paired HCES data were collected using questionnaires that included ≥100 foods.

### Estimation of input parameters for the LP analyses

The input model parameters, which were generated from the individual‐ and household‐level consumption data, included a list of available foods, a median daily portion size for each food (g/day), an upper and lower limit on the number of servings per week for each food, food subgroup, and food group, and a median number of servings per week for each food group (model goals). These parameters were generated, for each dataset, using a Microsoft® Access 2010 program developed to process dietary consumption data for analysis with Optifood.^38^

The food lists consisted of foods and beverages consumed by ≥5% of the households with a child aged 12–23 months (HCES data) or by the child (24HR data). Food or beverage items were excluded if they had no nutritional value (e.g., water, tea, and condiments) or, for the HCES data, if they would not typically be consumed by young children (e.g., alcoholic beverages). Breastmilk was included in all food lists.

The portion sizes (g/day), for each food in the food list from 24HR data, were median daily intakes for the consumers of each food. For breastmilk, the daily portion size was estimated by multiplying the recommended daily energy intake by 0.39 (recommended proportion of energy intake from breastmilk for 12‐ to 23‐month‐old children) and converting it to a gram weight assuming an energy content of 0.66 kcal/g.^39^ For HCES data, daily portion sizes for 12‐ to 23‐month‐old children were estimated using an AME quotient. Specifically, an AME for each household member was estimated by dividing their energy requirement (defined by age, sex, and pregnancy/lactation status if available) by the energy requirement of an adult male aged 18–29.9 years.^29^ The AME for a 12‐ to 23‐month‐old child was adjusted for recommended breastmilk intakes (i.e., AME was 61% of the median daily energy requirements).^37^ The AME quotient for a 12‐ to 23‐month‐old child was calculated by dividing their AME (adjusted for breastmilk) by the sum of AMEs across all household members. To estimate daily proportions of household foods consumed by a 12‐ to 23‐month‐old child, the household's food quantities were multiplied by the child's AME quotient and divided by the recall period of 7 or 14 days. The AMEs were estimated using the FAO/WHO/UNU energy requirements.^40^ When data on the lactation status of nonpregnant women were not available, we assumed the woman was breastfeeding if she had a child under 2 years of age. We also assumed that all children under 2 years of age were breastfed and infants aged less than 6 months of age were exclusively breastfed (i.e., did not consume household foods).

The lower and upper limits on the number of servings per week for each food subgroup and food group were defined as the 10th and 90th percentiles of consumption, and the food group goals were the 50th percentile for all children per dataset. For the 24HR datasets, the lower and upper limits on the number of servings per week for each food were based on the percentage of children in each dataset who had consumed it, as described elsewhere.^1^ In the HCES datasets, these limits were based on percentage of consuming households, as a proxy for percentage of children.

### Data analyses

All LP analyses were done using modules 1–3 in the Optifood software as described elsewhere.^1^, ^13^, ^41^ For each dataset pair, we used the same food composition tables (FCTs) to estimate the energy and nutrient content of modeled diets. These were the Nutrition Institute of Latin America and the Caribbean's (INCAP) FCT^42^ for Guatemalan datasets; the 2012 Harvest Plus FCT for Central and Eastern Uganda,^43^ for Ugandan datasets; the Indian National FCT and the U.S. Department of Agriculture (USDA) FCT^44^, ^45^ for the Bangladeshi datasets; and the USDA National Nutrient Database, Release 23,^45^ for Kenyan datasets. In all analyses, the FAO/WHO energy and recommended nutrient intakes (RNIs) were used to evaluate dietary adequacy and to define the module 2 model nutrient goals.^40^, ^46^, ^47^ The requirements for energy and protein were estimated using the WHO/FAO algorithms based on average body weight in each geographical area.^40^, ^47^ For all areas, because the consumption of animal‐source foods was low, we assumed the bioavailability of both zinc and iron was low.

“Problem nutrients” were defined as those that did not achieve 100% of their RNIs in the module 3–maximized diet (run without testing FBRs). The module 3–minimized diets simulate the lowest values in each nutrient's intake distribution. For these analyses, we defined a “minimized” model diet's nutrient content that met or exceeded 65% of the nutrient's RNI^1^, ^48^ as acceptable. Even though 65% of the RNI is less than the Estimated Average Requirement, it is widely used in Optifood analyses^1^ because it is expected to simulate a low proportion of the population at risk of inadequate intakes.^1^, ^15^

To standardize the module 3 analyses and enhance objectivity when making HCES and 24HR result comparisons, we (1) defined FBRs as the number of servings per week from individual food subgroups; (2) selected up to eight food subgroups to test as FBRs in module 3, which were those food subgroups that provided at least 5% of total nutrient content for the highest number of nutrients in the module 2 diet; (3) tested all possible combinations of these eight FBRs (i.e., up to 247 different sets of FBRs); (4) modeled the maximum number of servings per week allowed for each FBR (food subgroup) tested; and (5) selected the final set of FBRs for each data source and geographic area based on the combination with the highest number of minimized modeled diet nutrient contents that were ≥65% RNI using the lowest number of individual FBRs.

The LP inputs for each paired dataset were compared using the percentage interpair agreement or the ratio of HCES value divided by its paired individual 24HR value. Two methods were used to calculate the percentage interpair agreement, which were distinguished as the overall percentage agreement and the eligible percentage agreement. The overall percentage agreement was calculated as the number of matched items (i.e., paired HCES and 24HR values were the same) divided by the total number of these items across the paired HCES and 24HR datasets (i.e., in both or only one of the paired HCES or 24HR sets of input parameters) multiplied by 100. The overall percentage agreement was used to estimate interpair agreement for input model parameters (the food lists, food subgroups, and food groups modeled), assuming they should be the same in paired contexts, and for the classification of nutrients as “problem nutrients” or “adequate nutrients” (model outputs), as the nutrients modeled were identical. The eligible percentage agreement was calculated as the number of matched items divided by the number of items that occurred in both the paired HCES and 24HR analyses multiplied by 100. The eligible percentage agreement was used to estimate interpair agreement for model outputs (i.e., food subgroup sources of nutrients and FBRs) because results were dependent on the food lists modeled (e.g., the food subgroup “organ meats” could not be selected as a good source of nutrients or an FBR if there were no organ meats in the food list modeled, reflecting low availability/consumption in the population, despite being a potentially rich source of nutrients; infant cereals are another example of a food type that may not have appeared on both food lists, despite being consumed by infants, and require the use of both overall and eligible percentage agreement to capture the discrepancy).

## Results

### Dataset pairs

Eligible data pairs for 12‐ to 23‐month‐old children were identified for eight different areas across four countries from Latin America, South Asia, and East Africa (Table 1). Sample sizes ranged from 51 to 356 for 24HR data and from 31 to 227 for HCES data. The HCES food questionnaires included 116, 172, 196, and 299 foods in Guatemala, Uganda, Kenya, and Bangladesh, respectively.^49^, ^50^, ^51^, ^52^ The 24‐h dietary recall data were collected between 2012 and 2015 in two regions of Uganda, four counties of Kenya, one division in Bangladesh, and one region of Guatemala. The paired HCES datasets were collected between 2013 and 2016 in the same areas but not necessarily the same households. The year of data collection either aligned (Bangladesh) or differed by 1 to approximately 3 years. In most regions, the HCES data were collected over 12‐month periods, whereas the 24HR data were collected over 3‐ to 6‐month periods. The only area where data were collected over the same months of the year was in the Guatemalan Western Highlands, covering the harvest period. In Bangladesh, both HCES and 24HR data were collected during the “Rabi” winter season for sowing and growing in Sylhet even though the HCES data were collected over a longer period of time than the 24HR data.^53^, ^54^ Conversely, in the East African sites, HCES data were collected throughout the year, whereas 24HR data were collected at the end of the second rainy season and beginning of the second harvest in Uganda;^55^ during the long rains in Western Kenya (Vihiga); and at the end of the short rains harvest/beginning of long rains in the East and North Eastern Kenya (Kitui, Isiolo, and Marsabit).^56^

**Table 1:** Description of paired data by region, type of dietary data, source, sample size, and timing

Region, country	Type	Data source	*n*	Month and year
Western Highlands, Guatemala	24HR*^a^*	WFP/INCAP Nutrient Gap Study	246	August 2015–January 2016
	HCES*^b^*	Living Conditions Survey (ENCOVI)	227	August 2015–February 2016
Eastern Uganda, Uganda	24HR	ANI Nutrition Project Baseline	297	October–December 2014
	HCES	Uganda National Panel Study (Wave IV), UBOS	136	September 2013–August 2014
Western Uganda, Uganda	24HR	ANI Nutrition Project Baseline	356	October–December 2014
	HCES	Uganda National Panel Study (Wave IV), UBOS	118	September 2013–August 2014
Sylhet, Bangladesh	24HR	FAARM Baseline	51	January–April 2015
	HCES	Integrated Household Survey (BIHS)	119	January–July 2015
Isiolo, Kenya	24HR	REGAL IR Project	105	March–May 2013
	HCES	Integrated Household Budget Survey	40	September 2015–August 2016
Kitui, Kenya	24HR	Feed the Future Study	98	March–May 2012
	HCES	Integrated Household Budget Survey	42	September 2015–August 2016
Marsabit, Kenya	24HR	REGAL IR Project	139	March–May 2013
	HCES	Integrated Household Budget Survey	31	September 2015–August 2016
Vihiga, Kenya	24HR	Feed the Future Study	76	March–May 2012
	HCES	Integrated Household Budget Survey	36	September 2015–August 2016

^*a*^ 24‐h recall dietary data.

^*b*^ Household consumption and expenditure survey data.

### LP inputs (model parameters)

**Figure 1:**
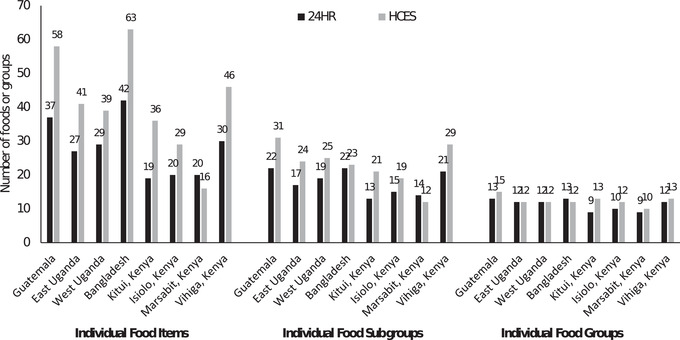
The mean number of foods, food subgroups, and food groups for 24‐h recall (24HR)–derived and household consumption and expenditure (HCES)–derived model parameters for analysis in Optifood by geographical area.

The numbers of foods and their corresponding food subgroups and food groups were generally higher in food lists generated using HCES data than 24HR data in all data pairs except for Marsabit, Kenya (foods and food subgroups; Fig. S1, online only) and Sylhet, Bangladesh (food groups; Fig. 1). The mean number of foods, food subgroups, and food groups modeled across the eight sites were 28, 18, and 11, respectively, for 24HR datasets, compared with 41, 23, and 12, respectively, for HCES datasets, which equated to ratios (HCES/24HR) of 0.8 (Marsabit), 1.3 (Western Uganda), 1.5 (Eastern Uganda, Isiolo, Vihiga, and Sylhet), 1.6 (Western Highlands), and 1.9 (Kitui). In contrast, the maximum food portion sizes were not consistently higher in the HCES than 24HR datasets. For matched foods, the median maximum portion size ratios (HCES/24HR) were <1 for three regions, 1.0 for two regions, and > 1 for three regions (Table 2), although it also depended on the food group examined (Table S1, online only). The HCES maximum food portion sizes were generally lower than 24HR portion sizes for foods in the added sugar (food portion ratio = 0.88), dairy products (0.81), fruits (0.94), and grains & grain products (0.85) food groups, and higher for foods in the added fats (1.09), bakery products (1.42), legumes, nuts & seeds (1.11), meat, fish & eggs (MFE; 1.01), starchy roots & other plant foods (1.28), and vegetables (1.18) food groups (Table S2, online only). The highest interpair maximum food portion size ratio agreement (i.e., percentage of ratios close to 1.0) was for foods in the added fat (25%), fruits (20%), and legumes, nuts & seeds (37.5%), food groups, whereas the lowest was for starchy roots & other plant foods (5.9%), MFE (14.3%), and vegetables (13.9%) (Table S2, online only).

**Table 2:** Summary of input model parameter agreement by geographical area

	Western Highlands, Guatemala	Eastern Uganda	Western Uganda	Sylhet, Bangladesh	Kitui, Kenya	Isiolo, Kenya	Marsabit, Kenya	Vihiga, Kenya	Mean of all values
Total number*^a^* of foods modeled	68	46	46	76	41	34	24	55	**48.8**
Total number of food subgroups modeled	31	26	28	27	21	23	15	30	**25.1**
Total number of food groups modeled	15	13	13	14	12	14	10	14	**13.1**
Median ratio maximum food portions*^b^*	1.00	1.19	1.39	1.14	0.62	0.78	0.54	1.00	**1.02** ***^d^***
% interpair overall agreement*^c^* foods modeled	39.7	45.7	47.8	38.2	34.1	44.1	50.0	38.2	**42.2**
% interpair overall agreement for food subgroups	71.0	57.7	57.1	66.7	61.9	47.8	73.3	66.7	**62.8**
% interpair overall agreement for food groups	86.7	84.6	84.6	78.6	83.3	57.1	90.0	78.6	**80.4**

^*a*^ Total number = number included in either the household consumption and expenditure survey (HCES) or 24‐h recall (24HR) paired models (i.e., included in either one or both paired HCES and 24HR models).

^*b*^ Ratio = HCES maximum weekly portion size (g/week)/24HR maximum weekly portion size (g/week).

^*c*^ % overall agreement = number included in both the paired HCES and 24HR models/the total number included across paired HCES and 24HR models (i.e., included in either one or both paired HCES and 24HR models).

^*d*^ Overall median value.

The mean interpair overall percentage agreement for matched foods, food subgroups, and food groups was 42% (range: 34–50%), 63% (range: 48–73%), and 80.4% (range: 57–90%), respectively (Table 2; Tables S1–S3, online only). The food subgroups for which interpair agreement was poor (<25%) were sugar‐sweetened beverages, broths or soups, and fortified margarine, whereas those within the food groups of dairy products, grains & grain products, starchy roots & other starchy plant foods, and vegetables generally showed perfect or good interpair agreement. Agreement for food subgroups within the MFE food group was mixed (i.e., poor to moderate; Table S3, online only). There was 100% matched interpair overall agreement for the food groups of added fats, added sugars, dairy products, grains & grain products, human milk, legumes, starchy roots & other starchy plant foods, and vegetables, but low interpair overall agreement (<15%) for sweetened snacks and desserts, miscellaneous, and beverages (Table S3, online only). When a food group was modeled in only one of the two HCES–24HR dataset pairs, it was included twice as often in the HCES as the 24HR set of food group model parameters. The discordant sets of food group model parameters occurred most often in Kitui, Kenya (Table S4, online only).

### LP outputs (model results)

The number of problem nutrients identified using the HCES‐generated model parameters was lower (three dataset pairs), the same as (four dataset pairs), or higher (one dataset pair) than those from the 24HR‐generated model parameters, although the mean overall percent agreement for problem nutrients was high at 95% (range: 77–100%; Table 3). Iron was a problem nutrient across all regions, and calcium and zinc for all but one region. The problem nutrients identified using 24HR‐ but not HCES‐derived model inputs were thiamine (three regions) or niacin (two regions), and using HCES‐derived inputs but not 24HR‐derived model inputs, they were calcium, vitamin A, and vitamin C (Marsabit; Table 3).

**Table 3:** The type and number of problem nutrients*^a^* and overall percentage agreements for nutrient classification by paired analyses and geographical area

	Western Highlands, Guatemala		Eastern Uganda		Western Uganda		Sylhet, Bangladesh		Kitui, Kenya		Isiolo, Kenya		Marsabit, Kenya		Vihiga, Kenya	
Nutrients	24HR*^c^*	HCES*^d^*	24HR	HCES	24HR	HCES	24HR	HCES	24HR	HCES	24HR	HCES	24HR	HCES	24HR	HCES
Fat																
Calcium			1	1	1	1	1	1	1	1	1	1		1	1	1
Folate			1	1			1	1					1	1		
Iron	1	1	1	1	1	1	1	1	1	1	1	1	1	1	1	1
Niacin			1						1	1	1	1	1	1	1	
Protein																
Riboflavin																
Thiamin			1						1		1	1			1	
Vitamin A														1		
Vitamin B12									1	1						
Vitamin B6													1	1		
Vitamin C														1		
Zinc			1	1	1	1	1	1	1	1	1	1	1	1	1	1
Number of problem nutrients	1	1	6	4	3	3	4	4	6	5	5	5	5	8	5	3
Overall agreement*^b^*, %	100		84.6		100		100		92.3		100		76.9		84.6	

^*a*^ Nutrients for which the modeled diet quantity was <100% of its recommended nutrient intake value in the module 3–maximized nutrient analyses.

^*b*^ Number of nutrients for which there was agreement (micronutrients identified or not identified as a problem nutrient across both data pairs), divided by total number of nutrients.

^*c*^ The number 1 indicates the nutrient was a problem nutrient when model parameters generated from individual 24‐h recall dietary data were used.

^*d*^ The number 1 indicates the nutrient was a problem nutrient when model parameters generated from household consumption and expenditure survey data were used.

The number of food subgroups providing ≥5% of at least one nutrient in the module 2–optimized diet ranged from 7 to 14, with a mean eligible percent agreement of 85% (range: 71–100%). The mean overall percent agreement for identifying food subgroup sources of nutrients was 81% (range: 71–100%; Table 4; Table S5, online only). Breastmilk, whole grains, and beans, lentils & peas were identified as good food subgroup sources of nutrients in all eight dataset pairs, and milk and green leafy vegetables were identified as good food subgroup sources of nutrients in seven of the eight data pairs (Table 4). The poorest overall agreement (≤50%) across geographical regions for individual food subgroups sources of nutrients was for soups/broths, organ meat, nuts & seeds, vitamin A–source vegetables, and vitamin C–rich vegetables, which partially reflects differences in the amounts and types of foods that were included in paired HCES and 24HR food lists (Table S5, online only). Agreement was moderate (51–75%) for refined grain bread, vitamin A–source fruit, vitamin C–rich fruit, eggs, fish without bones, red meat, other starchy plant foods, and other vegetables, and was good (>75%) for all other food subgroups modeled (Table S5, online only).

**Table 4:** Number of modeled nutrients for which each food subgroup was identified as a good nutrient source*^a^* and the percentage eligible and overall agreement between 24HR*^b^* and HCES*^c^* food list pairs by geographical area

	Western Highlands, Guatemala		Eastern Uganda		Western Uganda		Sylhet, Bangladesh		Kitui, Kenya		Isiolo, Kenya		Marsabit, Kenya		Vihiga, Kenya	
Food subgroups	24HR	HCES	24HR	HCES	24HR	HCES	24HR	HCES	24HR	HCES	24HR	HCES	24HR	HCES	24HR	HCES
Butter, ghee, margarine (unfortified)	.	0	.	.	0	0	.	.	.	0	0	0	0	0	0	0
Margarine (fortified)	.	.	.	0	0	0	.	.	2	.	0	.	0	.	0	0
Vegetable oil (fortified)	.	.	0	0	0	0	.	.	0	0	0	0	0	1	0	0
Vegetable oil (unfortified)	0	0	.	.			0	0	.	.	.	.	.	.	.	.
Sugar	0	0	0	0	0	0	0	0	0	0	0	0	0	0	0	0
Refined grain bread	0	5	6	8	6	6	.	.	.	0	.	0	.	.	0	1
Sweet bakery products	.	.	0	.	.	.	0	.	.	.	.	0	.	.	0	0
Sugar‐sweetened drinks	0	0	.	0	0	0	.	.	.	0	.	.	.	.	.	0
Broths or soups	0	2	0	.	.	.	.	.	4	.	.	.	.	.	0	.
Fluid or powdered milk	0	0	6	4	5	5	4	6	5	3	10	2	10	2	4	2
Other fruit	0	0	0	0	1	1	0	0	.	0	0	0	.	.	0	0
Vitamin A–source fruit	.	0	.	0	0	0	.	0	.	10	.	0	.	.	.	2
Vitamin C–rich fruit	0	0	0	1	0	0	1	1	.	0	.	.	.	.	0	1
Enriched/fortified grains and products	11	6	.	.	.	.	.	.	.	.	.	.	4	.	.	.
Refined grains and products (unfortified)	8	8	0	0	0	0	.	.	5	0	0	0	0	0	0	0
Whole grains and products (unfortified)	3	8	5	7	5	5	8	7	7	7	5	7	2	7	7	7
Breastmilk	10	10	10	10	10	10	10	10	10	10	10	10	10	10	10	10
Beans, lentils, peas	2	4	3	3	7	7	3	2	6	7	8	8	8	6	4	7
Nuts, seeds, not sweet	.	.	5	0	0	0	.	0	.	.	.	.	.	.	.	1
Eggs	2	2	.	8	0	0	1	0	.	0	0	.	.	.	.	0
Fish without bones	.	.	.	0	0	0	0	2	.	.	.	.	.	.	.	0
Organ meat	.	4	.	.	.	.	.	.	.		.	7	.	.	.	5
Pork	.	0	.	0	0	0	.	.	.	.	.	.	.	.	.	.
Poultry, rabbit	0	0	.	0	.	.	.	.	.	.	.	.	.	.	.	0
Red meat	.	0	.	2	7	7	.	.	.	1	.	3	5	8	2	2
Small, whole fish	.	.	6	7	0	0	3	2	.	.	.	.	.	.	7	2
Other composites	.	.	.	.	.	.	0	0	.	.	.	.	.	.	.	.
Other starchy plant foods	0	0	8	0	5	5	6	0	3	2	5	2	5	2	3	5
Condiment vegetables	0	0	0	0	0	0	0	1	.	.	.	.	.	.	.	0
Other vegetables	0	3	0	0	1	4	7	6	1	3	3	1	0	2	4	2
Vitamin A–source dark green leafy vegetables	5	5	8	3	7	6	7	9	10	8	7	5	.	.	9	10
Vitamin A–source other vegetables	0	0	0	2	0	0	.	.	.	.	.	.	.	.	1	0
Vitamin C–rich vegetables	0	0	1	2	7	7	0	7	6	0	.	1	2	.	0	0
No. of FSGs identified as good nutrient sources	7	11	10	12	11	11	10	11	11	9	7	10	8	8	10	14
No. of eligible FSG pairs	21		18		26		17		11		12		11		21	
No. of overall FSG pairs	26		28		26		20		22		20		14		30	
Eligible agreement*^d^*, %	85.7		77.8		100		70.6		81.8		100		81.8		85.7	
Overall agreement*^e^*, %	84.6		78.6		100		75.0		72.7		85.0		71.4		80.0	

^*a*^ Food subgroups were defined as a good source of a modeled nutrient if they provided ≥5% of that nutrient in the module 2, nutritionally best diet.

^*b*^ The analyses were done using model parameters generated from individual 24‐h recall dietary dataset.

^*c*^ The analyses were done using model parameters generated from household consumption and expenditure dietary dataset.

^*d*^ Data pairs agreed if food subgroups were a good source of at least one modeled nutrient across both dataset pairs or it was not a good source of any modeled nutrients for either dataset pair. Percentage agreement was calculated as the number of food subgroups for which there was agreement across both dataset pairs, divided by the number of eligible food subgroup pairs (i.e., food subgroups that were present in both paired datasets).

^*e*^ Data pairs agreed if food subgroups were a good source of at least one modeled nutrient across both dataset pairs or it was not a good source of any modeled nutrients for either dataset pair. Percentage agreement was calculated as the number of food subgroups for which there was agreement across both dataset pairs, divided by the number of food subgroups (i.e., food subgroups that were present in at least one of the paired datasets).

FSG, food subgroup.

**Figure 2:**
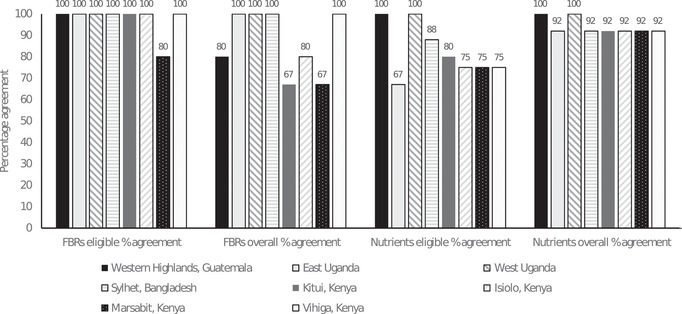
Eligible and overall percent agreement for the selection of food‐based recommendations (FBRs) and predictions of nutrients for which the population would likely be at risk of inadequate intake even if FBRs were adopted (module 3–minimized nutrient values <65% of their recommended nutrient intake values), when Optifood analyses were done using paired HCES‐ and 24HR‐derived input model parameters by geographical area.

Between six and eight food subgroups were tested as FBRs for each dataset (Table S6, online only). The final sets of FBRs selected included between three and six individual recommendations at the food subgroup level (Table S7, online only). Whole grain cereals, milk, and cooked beans, peas & lentils were included in most paired sets of recommendations (i.e., seven out of eight geographical areas), and vitamin A–source dark green leafy vegetables were included in paired sets of recommendations from six geographical areas. The mean overall percentage agreement for food subgroups included in paired HCES and 24HR sets of FBRs was 87% (range: 67–100%; Fig. 2). The mean eligible percentage agreement for food subgroups included in paired sets of FBRs was 97.5% (range: 80–100%). Eligible food subgroups were those tested in both the HCES and 24HR analyses.

The mean number of nutrients remaining below 65% of their RNIs when the final selected set of FBRs was tested (module 3, minimized nutrient content) was 3.5 (range: 1–8; Table S8, online only). Iron and zinc were low, for both HCES‐ and 24HR‐based models, in seven (zinc) or eight (iron) areas. The mean eligible percent agreement for identifying “inadequate nutrients” was high at 83% (range: 67–100%; Fig. 2). The mean overall percent agreement was also high at 94% (range: 92–100%). Across all geographical areas, the maximum interdataset difference in the number of “inadequate nutrients” was only one nutrient, and the overall percentage agreement was lowest for calcium (Table S8, online only).

## Discussion

Results from this study show that the overall percent agreements for HCES‐ and 24HR‐derived food subgroup and food group model input parameters were moderate to high, despite the relatively low overall percent agreement at the individual food level across all geographical areas. The higher number of foods in the HCESs compared with 24HR food lists (except for Marsabit) contributes to this low overall percent agreement at the food level, which may have been the result of differences in survey designs and the methods of assessment used in the paired data sources. HCESs were conducted over a 1‐year period compared with 2‐ to 6‐month periods for the 24HR surveys, which would capture to a greater extent than the 24HRs the seasonal variation in food availability and consumption. The higher percentage of seasonal fruits and vegetables in the HCES compared with 24HR food lists suggests that it was a contributing factor. The longer recall period of the HCES (i.e., 7–14 days) compared with the 24HR recall (i.e., 1 day) might also contribute to paired data‐source differences in food lists because it would capture less frequently consumed foods that may have been missed by a 24HR. Even though the closed, predefined food questionnaire used in an HCES might have missed some foods captured by the open, questioning approach of the 24HR, it also may have increased the number of foods recalled by reducing memory errors or introducing a social desirability bias, where participants report eating foods that were not consumed. The higher number of foods from the MFE food group recorded in the HCES than 24HR food lists suggests this might have occurred. Unfortunately, we cannot distinguish between modifiable factors (i.e., length of food questionnaire) and unmodifiable factors (i.e., assessment methods) that might have contributed to the low overall percent agreement at the model food list level. The length and specificity of food questionnaires in HCESs vary between countries and survey years,^28^ and given the requirements of the LP analysis,^13^ it is likely that surveys collecting data on less than 100 foods are not detailed enough for this type of analysis.

Other factors might have contributed to the low overall percent agreement at the food level, such as the age of the population modeled. Previous studies have shown that redistributed household‐level consumption data do not accurately estimate the food consumption of children under 2 years of age.^30^, ^34^, ^35^ The AME method used to estimate an individual's food intake from HCES data assumes that all family members consume the household's food supply in proportion to their energy requirements and that all food served to an individual was consumed, assumptions that might be incorrect for young children. Young children often consume fewer foods than adult family members; as they are learning to eat family foods,^39^ they may not finish all the food that they are served,^25^ and in some populations, they are fed special infant foods that would not be captured in an HCES.^30^, ^34^, ^35^ The higher percentage of foods from the nondairy beverages and MFE food groups and the lower percentage of foods from composite dishes in the modeled HCES than 24HR food list (Fig. S1, online only) suggest that age group might have influenced the overall percent agreement. A higher overall percent agreement might have been found comparing HCES and 24HR food lists if we had modeled an adult instead of a 12‐ to 23‐month‐old population.

Foods consumed outside the home are often underrepresented or overlooked when asking about household‐level consumption, compared with individual 24HRs.^25^, ^26^, ^28^ The proportion of foods consumed from restaurants, fast food outlets, street vendors, child care centers, schools, and workplaces is predicted to increase in LMICs as food systems evolve and purchasing power increases.^28^ Thus, for populations regularly consuming meals away from home (e.g., at childcare centers), depending on the types of foods consumed away from home, the overall % agreement might be lower than we found in these analyses. The addition of a “meals away from home module” that has been recommended by the nutrition community and adopted in some HCESs could overcome this issue by providing information on the types of foods consumed outside of home, the source, and who in the household is likely to have access to these foods.^28^, ^57^, ^58^, ^59^ The paired data‐source food list pattern observed in Marsabit was unique. Unlike the other seven geographical areas, the number of foods in the HCES food list was lower than in the 24HR list. Marsabit is an area of Kenya that experiences significant seasonal food insecurity and high poverty rates and has a large pastoralist population.^1^ The paired 24HR data used in this study were collected during the rainy season when the variety of nutritious foods is higher than in other seasons. This variety of food availability might have been captured to a greater extent in the 24HR data than HCES data, which were collected across the year. Alternatively, foods may have been reported in the 24HR but not in the HCES if, in food insecure households, young children are preferentially fed available food or families receive food aid for their children when levels of food insecurity are high. The fortified flour, animal milks, spaghetti, water gourd, and cabbage in the 24HR but not in the HCES food lists suggest that all three factors might have contributed to the pattern observed. Our results showing that context can influence the direction of difference in the number of foods derived from HCES‐ compared with 24HR‐generated food lists, at least for young children, is noteworthy; however, the overall percent agreement at the food level, even in this food insecure area, was similar to other regions (i.e., 50% in Marsabit versus 34–48% in the other regions). One advantage of using HCESs to develop FBRs with Optifood is that they usually collect data on food consumption across multiple seasons. This provides a good general understanding of the types of foods available across the year within each food subgroup, which is useful for developing FBRs at the food subgroup level, which may be more feasible to put into practice than FBRs focused on individual foods.

Despite this low overall percent agreement for HCES‐ and 24HR‐generated food lists, the overall percent agreement for food groups and food subgroups was moderate to high. More importantly, when examined by individual food groups and food subgroups, the overall percent agreement was only low for food groups of little nutritional importance (i.e., nondairy beverages, composites, bakery products, and miscellaneous), and it was high for nutritionally important food subgroups, such as small fish with bones, vitamin A–source dark green leafy vegetables, vitamin C–rich vegetables, milk, whole grain cereal, cooked beans, peas and lentils, and other fruit. This general pattern of agreement (i.e., paired‐method agreement for nutritious food groups/subgroup patterns) might account for the relatively high percent agreement of model outputs, including the identification of problem nutrients and good food subgroup sources of nutrients, the selection of FBRs, and predicting the nutritional benefits of adopting a set of FBRs. The sets of FBRs selected for young children across diverse geographical areas were similar, and most paired sets included the food subgroups of milk, whole grain cereals, cooked beans and lentils, and vitamin A–source dark green leafy vegetables. These results suggest that an important criterion when deciding on whether to use HCES data for an Optifood analyses is the extent to which food items from nutritious food subgroups are captured in its predefined food list. The critical nutritious food subgroups might also vary by age, sex, or physiological group because of differences in their nutrient requirements per unit energy intake. Thus, further analysis is recommended before extrapolating these promising results to other age or sex groups.

The approach we used to select and test FBRs in this study was more rigid than what would typically be followed in an Optifood analysis in order to reduce subjectivity when making paired data‐source (HCES versus 24HR) comparisons of generated results. This means that the final sets of FBRs presented here differ from those previously published using the same 24HR data.^1^, ^5^, ^60^ In practice, the modeling is carried out by or in partnership with stakeholders who either have knowledge of the local food system, dietary patterns, and FBR acceptability or who have specific policy or program applications in mind that would shape the analysis. Furthermore, prior to being promoted, we recommend that FBRs generated using Optifood are tested for feasibility and acceptability using tools, such as *Pro*PAN and Trials of Improved Practices, where caregivers are asked to trial and provide feedback on the recommendations.^13^, ^61^, ^62^, ^63^ This process of using local knowledge to select the final set of FBRs (i.e., a key model output) and field testing them would further minimize any differences in model outputs related to the sources of data used to define model parameters.

Nutrients we identified across all or almost all regions as problem nutrients (iron, zinc, calcium, and niacin) were consistent with published Optifood analyses that have been done for the same age group. Zinc, iron, and calcium were identified as problem nutrients in almost all (six out of seven) studies and niacin in half of the eligible studies.^1^, ^2^, ^3^, ^5^, ^8^, ^15^ This suggests that food systems in many LMICs may struggle to provide these nutrients in adequate amounts for 12‐ to 23‐month‐old children within acceptable dietary patterns. Tools, such as Optifood, can assist in identifying options for strengthening the dietary supply of these essential nutrients within the limitations of existing food systems.

The strengths of this study were the analyses of paired dataset from diverse geographical regions, including South Asia, Latin America, and Eastern Africa, and the inclusion of paired datasets from regions of Kenya experiencing marked differences in levels of food security. Also, we developed a method for processing HCES data that adjusts for breastmilk consumption, which addresses concerns raised about using HCES data to estimate the food consumption of children under 2 years of age.^30^ Our focus on young children was important given global interest in developing population‐specific FBRs for children 6‐ to 23‐months‐old to ameliorate the long‐term negative developmental and health impacts of undernutrition in this nutritionally vulnerable age group.^13^

A limitation of this study is that there is no gold standard against which HCES‐derived LP inputs can be compared. The 24HR is an imperfect method of dietary intake assessment that is prone to recall bias, inaccurate or imprecise quantity estimates, and short recall periods.^34^, ^64^, ^65^ In some respects, the use of HCES datasets has advantages over 24HR datasets for modeling in Optifood. They are routinely collected, nationally/regionally representative, based on large sample sizes, and capture data over a 12‐month period. At the household level, they also record information on the availability and consumption of a high number of foods over a 7‐ to 14‐day recall period to define model constraints on weekly food patterns (foods, food subgroups, and food groups) more accurately than a 1‐day 24HR. On the other hand, individual 24HRs have the advantage of being collected from the target groups of interest, which avoids potentially incorrect assumptions about intrahousehold distribution of food when estimating individual intakes from HCES household food consumption data. Another limitation is the paired 24HR and HCES datasets were not collected over the same period of time. This limitation would decrease overall percent agreements. The level of agreement might be higher than those reported in this study if data were collected at the same time, especially at the food level. After excluding households without a 12‐ to 23‐month‐old child, HCES sample sizes for the four geographic regions in Kenya were less than the recommended minimum of 50 individuals for Optifood analysis using 24HR data to provide adequate data points for determining food portion sizes.^1^ As such, HCES Optifood inputs for these areas could be less reliable than data collected from a larger sample. However, this may be less relevant as HCES data collection focuses on foods that are already established to be commonly consumed and as food consumption is reported at the household level. As these inputs were based on redistributed household‐level data, reflecting foods that are commonly available and consumed by households in general rather than the specific target group, further analysis could test the utility of consumption data from a wider selection of households in HCES datasets (e.g., expanding the definition to households with children <5 years old) in cases where sample sizes were insufficient.

The LP‐based Optifood tool can objectively and rapidly inform both nutrition‐specific and ‐sensitive programming. It has been successfully applied to identify problem nutrients in local food systems, develop population‐specific FBRs, test the nutrition potential of existing or proposed dietary recommendations, and inform the design of other intervention strategies, such as the use of special fortified foods, multiple micronutrient powders, or lipid‐based nutrient supplements.^1^, ^3^, ^4^, ^5^, ^6^, ^16^, ^60^, ^66^, ^67^ While the requirement for individual dietary recall data has limited opportunities for governmental and nongovernmental organizations to use Optifood to generate context‐specific evidence to inform programmatic or policy decisions, this study suggests that household‐level HCES data can replace individual‐level dietary data, when the latter is not available, to generate evidence for program guidance. Furthermore, the risks of using HCES instead of individual dietary data to formulate FBRs are minimized by active stakeholder engagement in the modeling process and qualitative field testing of proposed sets of FBRs before adopting them for programmatic use. This opportunity is noteworthy because HCESs are routinely collected, generally publicly accessible, and representative at the national and regional level for over 116 countries.^36^ The utility of HCES household consumption data, for nutrition analyses, has also improved markedly over the last 5–10 years. It should continue to improve given the current work program that is focused on strengthening the overall quality of HCES data and the measurement of food consumption.^25^, ^28^, ^68^ Based on our results, government bodies will be able in the future to use HCES‐generated model parameters in Optifood to rapidly generate evidence that informs policy questions related to strengthening local food systems and providing guidance on food‐based strategies for improving the dietary adequacy of vulnerable populations.

## Author contributions

F.K., E.F., M.W., K.S., and G.B. conceptualized the analyses. F.K. and E.F. performed the analyses, interpreted the results, and take responsibility for the integrity of the data analyzed. F.K. and E.F. wrote the manuscript. All other authors read and approved the final manuscript.

## Competing interests

The authors declare no competing interests.

### Peer review

The peer review history for this article is available at https://publons.com/publon/10.1111/nyas.14709.

## Supporting information


**Figure S1**. Percent of foods within each food group that was only reported in the HCES‐derived food list, the 24‐h‐recall–derived food list, and in both datasets across all eight geographical areas.
**Table S1**. Maximum amount each food was modeled (g/week) and the ratio of these amounts by dataset type, region, and food group.
**Table S2**. Number of matched maximum food portions (g/week), median ratio of maximum food portions, the percentage of ratios that were within given limits by selected food groups, and the percentage of HCES maximum food portions that were higher than 24‐h maximum food portions.
**Table S3**. Agreement in the food groups and food subgroups modeled between paired 24HR‐ and HCES‐derived model parameters, by geographical area.
**Table S4**. Summary of agreement between paired 24HR‐ and HCES‐derived model parameters at the food group level.
**Table S5**. Number of modeled nutrients for which each food subgroup was identified as a good nutrient source and the percentage eligible and overall agreement between 24HR and HCES food list pairs, by geographical area.
**Table S6**. Draft individual food‐based recommendations (expressed as the number of average portions per food subgroup per week) tested (module 3, minimized analyses), by geographic area and data source.
**Table S7**. Final food‐based recommendations (FBRs) selected (expressed as grams per week), by geographical area
**Table S8**. Nutrients that remained below 65% of the recommended levels when the final sets of food‐based recommendations were tested (module 3, minimized nutrient values) and data pair percent agreements.
