# Supplementary material for: Household‐level consumption data can be redistributed for individual‐level Optifood diet modeling: analysis from four countries
Source: Ann N Y Acad Sci. 2021 Nov 30;1509(1):145–60. doi: 10.1111/nyas.14709 (PMC9299870; doi:10.1111/nyas.14709)
Supplement: Supplementary file 1 — Figure S1. Percent of foods within each food group that was only reported in the HCES‐derived food list, the 24‐h‐recall–derived food list, and in both datasets across all eight geographical areas. Table S1. Maximum amount each food was modeled (g/week) and the ratio of these amounts by dataset type, region, and food group. Table S2. Number of matched maximum food portions (g/week), median ratio of maximum food portions, the percentage of ratios that were within given limits by selected food groups, and the percentage of HCES maximum food portions that were higher than 24‐h maximum food portions. Table S3. Agreement in the food groups and food subgroups modeled between paired 24HR‐ and HCES‐derived model parameters, by geographical area. Table S4. Summary of agreement between paired 24HR‐ and HCES‐derived model parameters at the food group level. Table S5. Number of modeled nutrients for which each food subgroup was identified as a good nutrient source and the percentage eligible and overall agreement between 24HR and HCES food list pairs, by geographical area. Table S6. Draft individual food‐based recommendations (expressed as the number of average portions per food subgroup per week) tested (module 3, minimized analyses), by geographic area and data source. Table S7. Final food‐based recommendations (FBRs) selected (expressed as grams per week), by geographical area Table S8. Nutrients that remained below 65% of the recommended levels when the final sets of food‐based recommendations were tested (module 3, minimized nutrient values) and data pair percent agreements. [file NYAS-1509-145-s001.docx]

**Table S1:** Maximum amount each food was modelled (g/week)^1^ and the ratio of these amounts^2^ by dataset type, region and food group

| **Food** | **Western Highlands** | | | **East Uganda** | | | **West Uganda** | | | **Sylhet** | | | **Kitui** | | | **Isiolo** | | | **Marsabit** | | | **Vihiga** | | |
| --- | --- | --- | --- | --- | --- | --- | --- | --- | --- | --- | --- | --- | --- | --- | --- | --- | --- | --- | --- | --- | --- | --- | --- | --- |
|  | 24HR^3^ | HCES^4^ | Ratio^5^ | 24HR | HCES | Ratio | 24HR | HCES | Ratio | 24HR | HCES | Ratio | 24HR | HCES | Ratio | 24HR | HCES | Ratio | 24HR | HCES | Ratio | 24HR | HCES | Ratio |
| **Added Fats** | | | | | | | | | | | | | | | | | | | | | | | | |
| Ghee, butter |  |  |  |  |  |  |  | 16 |  |  |  |  |  | 21 |  |  |  |  |  |  |  |  |  |  |
| Margarine, F^5^ |  |  |  |  | 8 |  |  | 14 |  |  |  |  | 42 |  |  | 20 |  |  | 15 |  |  | 6 | 7 | **1.17** |
| Margarine, UF^4^ |  | 20 |  |  |  |  |  |  |  |  |  |  |  | 21 |  | 20 | 14 | **0.70** | 32 | 14 | **0.44** | 10 | 14 | **1.40** |
| Mustard oil |  |  |  |  |  |  |  |  |  |  | 34 |  |  |  |  |  |  |  |  |  |  |  |  |  |
| Soybean oil |  |  |  |  |  |  |  |  |  | 76 | 78 | **1.02** |  |  |  |  |  |  |  |  |  |  |  |  |
| Vegetable oil, F |  |  |  | 14 | 20 | **1.43** | 14 | 20 | **1.43** |  |  |  | 8 | 70 | **8.75** | 27 | 25 | **0.93** | 65 | 35 | **0.54** | 14 | 14 | **1.00** |
| Vegetable oil, UF | 24 | 60 | **2.52** |  |  |  |  |  |  |  |  |  |  |  |  |  |  |  |  |  |  |  |  |  |
| **Added Sugars** | | | | | | | | | | | | | | | | | | | | | | | | |
| Honey, syrup |  |  |  |  |  |  |  |  |  |  |  |  |  | 28 |  |  |  |  |  |  |  |  |  |  |
| Gur |  |  |  |  |  |  |  |  |  |  | 44 |  |  |  |  |  |  |  |  |  |  |  |  |  |
| Jaggery |  |  |  |  |  |  |  |  |  |  |  |  |  |  |  |  |  |  |  |  |  |  |  |  |
| Sugar | 281 | 60 | **0.21** | 70 | 104 | **1.49** | 84 | 96 | **1.14** | 70 | 73 | **1.04** | 70 | 42 | **0.60** | 126 | 91 | **0.72** | 98 | 28 | **0.29** | 77 | 98 | **1.27** |
| **Bakery Products** | | | | | | | | | | | | | | | | | | | | | | | | |
| Biscuit, sweet, plain |  |  |  |  |  |  |  |  |  | 39 |  |  |  |  |  |  | 77 |  |  |  |  |  | 63 |  |
| Bread, other |  |  |  |  | 185 |  |  |  |  |  |  |  |  |  |  |  |  |  |  |  |  |  | 119 |  |
| Bread, refined | 60 |  |  | 124 | 176 | **1.42** | 151 | 205 | **1.36** |  |  |  |  | 49 |  |  | 7 |  |  |  |  | 40 | 56 | **1.40** |
| Bread, sweet | 68 | 106 | **1.56** |  |  |  |  |  |  |  |  |  |  |  |  |  |  |  |  |  |  |  |  |  |
| Cake |  |  |  |  |  |  |  |  |  | 51 |  |  |  |  |  |  |  |  |  |  |  |  |  |  |
| Chapati |  |  |  | 60 | 156 | **2.60** |  | 82 |  |  |  |  |  |  |  |  |  |  |  |  |  |  |  |  |
| French bread |  | 130 |  |  |  |  |  |  |  |  |  |  |  |  |  |  |  |  |  |  |  |  |  |  |
| Mandazi donut |  |  |  | 39 |  |  |  |  |  |  |  |  |  |  |  |  |  |  |  |  |  | 200 |  |  |
| **Beverages** (non-dairy) | | | | | | | | | | | | | | | | | | | | | | | | |
| Carbonated soda | 100 | 265 | **2.65** |  | 79 |  |  | 96 |  |  |  |  |  | 14 |  |  |  |  |  |  |  |  | 49 |  |
| Chocolate powder |  |  |  |  |  |  |  |  |  |  |  |  |  |  |  |  |  |  |  |  |  |  | 7 |  |
| Fruit beverage from powder | 100 |  |  |  |  |  |  |  |  |  |  |  |  |  |  |  |  |  |  |  |  |  |  |  |
| Fruit Juice |  | 198 |  |  |  |  |  |  |  |  | 28 |  |  |  |  |  |  |  |  |  |  |  |  |  |
| Soymilk |  |  |  |  |  |  |  |  |  |  |  |  |  |  |  |  |  |  |  | 49 |  |  |  |  |
| **Composites** | | | | | | | | | | | | | | | | | | | | | | | | |
| Beef broth or soup |  |  |  | 70 |  |  | 74 |  |  |  |  |  | 111 |  |  |  |  |  |  |  |  |  |  |  |
| Chicken soup & noodles, dehydrated | 21 |  |  |  |  |  |  |  |  |  |  |  |  |  |  |  |  |  |  |  |  |  |  |  |
| Chicken soup, dehydrated | 7 | 19 | **2.77** |  |  |  |  |  |  |  |  |  |  |  |  |  |  |  |  |  |  |  |  |  |
| Fish broth or soup |  |  |  | 60 |  |  | 58 |  |  |  |  |  |  |  |  |  |  |  |  |  |  | 72 |  |  |
| Vegetable broth |  |  |  |  |  |  |  |  |  |  |  |  | 553 |  |  |  |  |  |  |  |  | 237 |  |  |
| Cassava and millet porridge |  |  |  |  |  |  | 30 |  |  |  |  |  |  |  |  |  |  |  |  |  |  |  |  |  |
| Bombay mix |  |  |  |  |  |  |  |  |  | 30 |  |  |  |  |  |  |  |  |  |  |  |  |  |  |
| Rice snack, savory |  |  |  |  |  |  |  |  |  | 40 |  |  |  |  |  |  |  |  |  |  |  |  |  |  |
| Rice snack, sweet |  |  |  |  |  |  |  |  |  |  | 90 |  |  |  |  |  |  |  |  |  |  |  |  |  |
| **Dairy Products** | | | | | | | | | | | | | | | | | | | | | | | | |
| Fresh cheese | 21 | 29 | **1.39** |  |  |  |  |  |  |  |  |  |  |  |  |  |  |  |  |  |  |  |  |  |
| Fresh cream |  | 127 |  |  |  |  |  |  |  |  |  |  |  |  |  |  |  |  |  |  |  |  |  |  |
| Camel milk, fresh |  |  |  |  |  |  |  |  |  |  |  |  |  |  |  |  | 21 |  | 2254 |  |  |  |  |  |
| Cow milk, fermented |  |  |  |  |  |  |  |  |  |  |  |  |  | 175 |  |  |  |  |  |  |  |  | 35 |  |
| Cow milk, fresh |  | 154 |  | 420 | 400 | **0.95** | 511 | 504 | **0.99** | 330 | 344 | **1.04** | 428 | 84 | **0.20** | 253 | 168 | **0.66** | 621 | 182 | **0.29** | 406 | 189 | **0.47** |
| **Food** (cont.) | **Western Highlands** | | | **East Uganda** | | | **West Uganda** | | | **Sylhet** | | | **Kitui** | | | **Isiolo** | | | **Marsabit** | | | **Vihiga** | | |
|  | 24HR^3^ | HCES^4^ | Ratio^5^ | 24HR | HCES | Ratio | 24HR | HCES | Ratio | 24HR | HCES | Ratio | 24HR | HCES | Ratio | 24HR | HCES | Ratio | 24HR | HCES | Ratio | 24HR | HCES | Ratio |
| Goat milk, fresh |  |  |  |  |  |  |  |  |  |  |  |  | 165 |  |  | 920 |  |  | 620 |  |  |  |  |  |
| Powdered milk | 42 | 13 | **0.31** |  |  |  |  |  |  | 4 | 32 | **8.03** |  |  |  |  | 7 |  |  |  |  |  |  |  |
| **Fruits** | | | | | | | | | | | | | | | | | | | | | | | | |
| Apple | 162 | 50 | **0.31** |  |  |  |  |  |  | 135 | 77 | **0.57** |  |  |  |  |  |  |  |  |  |  |  |  |
| Banana, ripe | 330 | 292 | **0.88** |  | 364 |  |  | 392 |  | 33 | 150 | **4.55** |  | 56 |  | 110 | 128 | **1.16** |  |  |  | 232 | 224 | **0.97** |
| Grapes |  |  |  |  |  |  |  |  |  |  | 58 |  |  |  |  |  |  |  |  |  |  |  |  |  |
| Guava |  |  |  |  |  |  |  |  |  |  |  |  |  |  |  |  |  |  |  |  |  | 150 |  |  |
| Jackfruit |  |  |  | 108 | 156 | **1.44** | 48 |  |  |  | 348 |  |  |  |  |  |  |  |  |  |  |  |  |  |
| Jujube |  |  |  |  |  |  |  |  |  | 129 | 78 | **0.60** |  |  |  |  |  |  |  |  |  |  |  |  |
| Lemon | 30 | 40 | **1.33** |  |  |  |  |  |  |  | 55 |  |  |  |  |  |  |  |  |  |  |  | 77 |  |
| Mandarin |  | 40 |  |  |  |  |  |  |  |  |  |  |  |  |  |  |  |  |  |  |  |  |  |  |
| Mango |  |  |  |  | 1276 |  |  | 212 |  |  | 182 |  |  | 1302 |  |  | 21 |  |  |  |  |  | 357 |  |
| Mango (green) |  |  |  |  |  |  |  |  |  |  | 130 |  |  |  |  |  |  |  |  |  |  |  |  |  |
| Orange | 100 | 92 | **0.92** |  | 532 |  |  |  |  |  |  |  |  | 77 |  |  |  |  |  |  |  |  | 98 |  |
| Papaya |  | 98 |  |  | 150 |  |  | 208 |  |  | 174 |  |  | 56 |  |  |  |  |  |  |  |  | 70 |  |
| Passionfruit |  |  |  |  | 36 |  |  | 44 |  |  |  |  |  |  |  |  |  |  |  |  |  |  |  |  |
| Watermelon |  | 100 |  |  |  |  |  |  |  |  | 300 |  |  |  |  |  |  |  |  |  |  |  |  |  |
| **Grains & Grain Products** | | | | | | | | | | | | | | | | | | | | | | | | |
| Atta flour, white |  |  |  |  |  |  |  |  |  | 294 | 143 | **0.49** |  |  |  |  |  |  |  |  |  |  |  |  |
| Breakfast cereals |  | 54 |  |  |  |  |  |  |  |  |  |  |  |  |  |  |  |  |  |  |  |  |  |  |
| Incaparina | 112 | 41 | **0.37** |  |  |  |  |  |  |  |  |  |  |  |  |  |  |  |  |  |  |  |  |  |
| Maize | 945 | 1058 | **1.12** |  |  |  |  |  |  |  |  |  |  |  |  |  |  |  |  |  |  |  |  |  |
| Maize flour, F |  |  |  |  |  |  |  |  |  |  |  |  |  |  |  |  |  |  | 104 |  |  |  |  |  |
| Maize flour, white |  |  |  | 560 | 485 | **0.87** | 800 | 612 | **0.77** |  |  |  | 434 | 280 | **0.65** | 315 | 410 | **1.30** | 284 | 154 | **0.54** | 399 | 427 | **1.07** |
| Maize whole, white |  |  |  |  |  |  |  |  |  |  |  |  | 420 | 623 | **1.48** |  | 315 |  |  | 245 |  |  | 399 |  |
| Maize whole, yellow |  |  |  | 1000 | 1365 |  | 2590 | 872 | **0.34** |  |  |  |  |  |  |  |  |  |  |  |  |  |  |  |
| Millet flour |  |  |  | 700 | 777 | **1.11** | 500 | 420 | **0.84** |  |  |  | 61 | 42 | **0.69** |  |  |  |  |  |  | 42 | 35 | **0.83** |
| Noodles |  |  |  |  |  |  |  |  |  |  | 55 |  |  |  |  |  |  |  |  |  |  |  |  |  |
| Oatmeal | 20 | 62 | **3.11** |  |  |  |  |  |  |  |  |  |  |  |  |  |  |  |  |  |  |  |  |  |
| Oatmeal, F | 15 |  |  |  |  |  |  |  |  |  |  |  |  |  |  |  |  |  |  |  |  |  |  |  |
| Other atoles |  | 57 |  |  |  |  |  |  |  |  |  |  |  |  |  |  |  |  |  |  |  |  |  |  |
| Puffed Rice |  |  |  |  |  |  |  |  |  |  | 64 |  |  |  |  |  |  |  |  |  |  |  |  |  |
| Rice bran |  |  |  |  |  |  |  |  |  | 160 |  |  |  |  |  |  |  |  |  |  |  |  |  |  |
| Rice, fine |  |  |  |  |  |  |  |  |  |  | 991 |  |  |  |  |  |  |  |  |  |  |  |  |  |
| Rice, parboiled |  |  |  |  |  |  |  |  |  | 811 | 862 | **1.06** |  |  |  |  |  |  |  |  |  |  |  |  |
| Rice, white, UF | 196 | 57 | **0.29** | 175 | 208 | **1.19** | 150 | 172 | **1.15** | 242 | 1077 | **4.46** | 224 | 49 | **0.22** | 357 | 220 | **0.62** | 210 | 238 | **1.13** | 81 | 49 | **0.60** |
| Rice, white, F | 26 |  |  |  |  |  |  |  |  |  |  |  |  |  |  |  |  |  |  |  |  |  |  |  |
| Sorghum flour |  |  |  |  | 279 |  |  |  |  |  |  |  | 44 | 60 | **1.36** |  |  |  |  |  |  | 24 | 35 | **1.46** |
| Spaghetti | 105 | 29 | **0.28** |  |  |  |  |  |  |  |  |  |  |  |  |  |  |  | 100 |  |  |  |  |  |
| Tamales |  | 165 |  |  |  |  |  |  |  |  |  |  |  |  |  |  |  |  |  |  |  |  |  |  |
| Tortillas | 315 | 1031 | **3.27** |  |  |  |  |  |  |  |  |  |  |  |  |  |  |  |  |  |  |  |  |  |
| Wheat flour, refined |  |  |  |  |  |  |  |  |  |  |  |  |  | 7 |  |  | 7 |  | 74 | 7 | **0.10** |  | 7 |  |
| **Human milk** | | | | | | | | | | | | | | | | | | | | | | | | |
| Breastmilk | 3564 | 3564 | **1.00** | 3152 | 3152 | **1.00** | 3145 | 3145 | **1.00** | 3117 | 3117 | **1.00** | 3176 | 3176 | **1.00** | 3070 | 3070 | **1.00** | 3007 | 3007 | **1.00** | 3449 | 3449 | **1.00** |
| **Legumes** | | | | | | | | | | | | | | | | | | | | | | | | |
| Beans, black | 84 | 84 | **1.00** |  |  |  |  |  |  |  |  |  |  |  |  |  |  |  |  |  |  |  |  |  |
| Beans, cranberry |  |  |  | 150 |  |  |  |  |  |  |  |  |  |  |  | 64 |  |  |  |  |  |  |  |  |
| **Food** (cont.) | **Western Highlands** | | | **East Uganda** | | | **West Uganda** | | | **Sylhet** | | | **Kitui** | | | **Isiolo** | | | **Marsabit** | | | **Vihiga** | | |
|  | 24HR^3^ | HCES^4^ | Ratio^5^ | 24HR | HCES | Ratio | 24HR | HCES | Ratio | 24HR | HCES | Ratio | 24HR | HCES | Ratio | 24HR | HCES | Ratio | 24HR | HCES | Ratio | 24HR | HCES | Ratio |
| Beans, kidney |  |  |  | 57 |  |  | 18 |  |  |  |  |  | 105 |  |  | 84 |  |  | 90 |  |  |  |  |  |
| Beans, navy |  |  |  |  |  |  | 184 |  |  |  |  |  |  |  |  |  |  |  |  |  |  |  |  |  |
| Beans, pinto |  |  |  |  | 156 |  | 176 | 176 | **1.00** |  |  |  |  | 126 |  | 147 | 98 | **0.67** | 140 | 147 | **1.05** | 81 | 119 | **1.47** |
| Broadbeans | 20 |  |  |  |  |  |  |  |  |  |  |  |  |  |  |  |  |  |  |  |  |  |  |  |
| Chickpeas |  |  |  |  |  |  |  |  |  | 30 |  |  |  |  |  |  |  |  |  |  |  |  |  |  |
| Cowpeas |  |  |  |  |  |  |  |  |  |  |  |  |  | 49 |  |  |  |  |  |  |  |  |  |  |
| Flat bean seeds |  |  |  |  |  |  |  |  |  |  | 77 |  |  |  |  |  |  |  |  |  |  |  |  |  |
| Groundnuts |  |  |  | 60 | 70 | **1.17** | 75 | 102 | **1.36** |  |  |  |  |  |  |  |  |  |  |  |  |  | 21 |  |
| Jackfruit Seed |  |  |  |  |  |  |  |  |  |  | 144 |  |  |  |  |  |  |  |  |  |  |  |  |  |
| Lentils |  |  |  |  |  |  |  |  |  | 36 | 44 | **1.21** |  |  |  |  |  |  |  |  |  |  |  |  |
| Peanut butter |  |  |  |  | 91 |  |  |  |  |  |  |  |  |  |  |  |  |  |  |  |  |  |  |  |
| Soybeans |  |  |  |  |  |  | 65 |  |  |  |  |  |  |  |  |  |  |  |  |  |  |  |  |  |
| Split peas |  |  |  |  |  |  |  |  |  |  |  |  |  | 56 |  |  | 91 |  |  |  |  |  |  |  |
| **Meat, Fish & Eggs** | | | | | | | | | | | | | | | | | | | | | | | | |
| Egg, chicken | 108 | 110 | **1.01** |  | 285 |  | 172 | 192 | **1.12** | 90 | 51 | **0.57** |  | 56 |  | 12 |  |  |  |  |  |  | 112 |  |
| Egg, duck |  |  |  |  |  |  |  |  |  | 30 |  |  |  |  |  |  |  |  |  |  |  |  |  |  |
| Fish, climbing perch |  |  |  |  |  |  |  |  |  | 36 |  |  |  |  |  |  |  |  |  |  |  |  |  |  |
| Fish, grass carp |  |  |  |  |  |  |  |  |  |  | 19 |  |  |  |  |  |  |  |  |  |  |  |  |  |
| Fish, mrigal |  |  |  |  |  |  |  |  |  |  | 21 |  |  |  |  |  |  |  |  |  |  |  |  |  |
| Fish, pangash |  |  |  |  |  |  |  |  |  |  | 41 |  |  |  |  |  |  |  |  |  |  |  |  |  |
| Fish, rui/rohu |  |  |  |  |  |  |  |  |  |  | 30 |  |  |  |  |  |  |  |  |  |  |  |  |  |
| Fish, silver carp |  |  |  |  |  |  |  |  |  | 24 |  |  |  |  |  |  |  |  |  |  |  |  |  |  |
| Fish, spotted snakehead |  |  |  |  |  |  |  |  |  | 26 | 32 | **1.23** |  |  |  |  |  |  |  |  |  |  |  |  |
| Fish, tilapia |  |  |  |  | 96 |  |  | 120 |  | 11 | 26 | **2.46** |  |  |  |  |  |  |  |  |  |  | 42 |  |
| Liver, chicken |  | 20 |  |  |  |  |  |  |  |  |  |  |  |  |  |  | 35 |  |  |  |  |  | 21 |  |
| Meat, pork |  | 21 |  |  | 39 |  |  | 104 |  |  |  |  |  |  |  |  |  |  |  |  |  |  |  |  |
| Meat, chicken | 66 | 25 | **0.38** |  | 30 |  |  |  |  |  | 37 |  |  |  |  |  |  |  |  |  |  |  | 28 |  |
| Meat, processed |  | 49 |  |  |  |  |  |  |  |  |  |  |  |  |  |  |  |  |  |  |  |  |  |  |
| Meat, beef |  | 25 |  |  | 41 |  | 26 | 102 | **3.92** |  | 27 |  |  | 35 |  |  | 35 |  |  | 28 |  | 38 | 42 | **1.11** |
| Meat, goat |  |  |  |  |  |  |  |  |  |  |  |  |  | 14 |  |  | 28 |  | 74 | 525 | **7.09** |  |  |  |
| Prawn |  |  |  |  |  |  |  |  |  | 30 |  |  |  |  |  |  |  |  |  |  |  |  |  |  |
| Small fish, karfu |  |  |  |  |  |  |  |  |  | 15 |  |  |  |  |  |  |  |  |  |  |  |  |  |  |
| Small fish, khalisa |  |  |  |  |  |  |  |  |  |  | 23 |  |  |  |  |  |  |  |  |  |  |  |  |  |
| Small fish, Mola |  |  |  |  |  |  |  |  |  |  | 13 |  |  |  |  |  |  |  |  |  |  |  |  |  |
| Small fish, panch mishali |  |  |  |  |  |  |  |  |  | 60 | 20 | **0.33** |  |  |  |  |  |  |  |  |  |  |  |  |
| Small fish, puti |  |  |  |  |  |  |  |  |  | 20 | 13 | **0.65** |  |  |  |  |  |  |  |  |  |  |  |  |
| Small fish, dried |  |  |  | 84 | 85 | **1.01** |  | 112 |  | 10 | 5 | **0.48** |  |  |  |  |  |  |  |  |  | 80 | 28 | **0.35** |
| **Roots & Tubers** | | | | | | | | | | | | | | | | | | | | | | | | |
| Banana, green |  | 496 |  | 244 | 162 | **0.66** | 260 | 368 | **1.42** |  |  |  |  | 105 |  | 180 | 110 | **0.61** |  |  |  | 354 | 35 | **0.10** |
| Cassava flour |  |  |  | 225 | 760 | **3.38** | 210 | 488 | **2.32** |  |  |  |  | 56 |  |  |  |  |  |  |  | 210 | 196 | **0.93** |
| Cassava, fresh |  |  |  |  | 280 |  | 164 | 476 | **2.90** |  |  |  |  |  |  |  |  |  |  |  |  |  |  |  |
| Irish Potato | 245 | 340 | **1.39** |  | 156 |  | 308 | 618 | **2.01** | 210 | 269 | **1.28** | 212 | 42 | **0.20** | 343 | 267 | **0.78** | 315 | 42 | **0.13** | 200 | 161 | **0.81** |
| Sweet potato, white |  |  |  | 630 | 828 | **1.31** | 244 | 702 | **2.88** |  |  |  |  |  |  |  |  |  |  |  |  |  | 196 |  |
| **Sweets** | | | | | | | | | | | | | | | | | | | | | | | | |
| Candies |  | 13 |  |  |  |  |  |  |  |  |  |  |  | 91 |  |  | 126 |  |  |  |  |  | 259 |  |
| Chocolate |  |  |  |  |  |  |  |  |  | 10 |  |  |  |  |  |  |  |  |  |  |  |  |  |  |
| **Food** (cont.) | **Western Highlands** | | | **East Uganda** | | | **West Uganda** | | | **Sylhet** | | | **Kitui** | | | **Isiolo** | | | **Marsabit** | | | **Vihiga** | | |
|  | 24HR^3^ | HCES^4^ | Ratio^5^ | 24HR | HCES | Ratio | 24HR | HCES | Ratio | 24HR | HCES | Ratio | 24HR | HCES | Ratio | 24HR | HCES | Ratio | 24HR | HCES | Ratio | 24HR | HCES | Ratio |
| **Sauces** | | | | | | | | | | | | | | | | | | | | | | | | |
| Mayonnaise |  | 20 |  |  |  |  |  |  |  |  |  |  |  |  |  |  |  |  |  |  |  |  |  |  |
| **Vegetables** | | | | | | | | | | | | | | | | | | | | | | | | |
| Amaranth |  |  |  | 80 | 76 | **0.95** | 98 | 147 | **1.50** | 105 | 120 | **1.14** |  |  |  |  |  |  |  |  |  |  |  |  |
| Apazote | 2 |  |  |  |  |  |  |  |  |  |  |  |  |  |  |  |  |  |  |  |  |  |  |  |
| Ash gourd |  |  |  |  |  |  |  |  |  |  | 240 |  |  |  |  |  |  |  |  |  |  |  |  |  |
| Avocado |  | 42 |  |  | 48 |  |  | 680 |  |  |  |  |  | 84 |  |  | 49 |  |  |  |  | 168 | 126 | **0.75** |
| Bitter gourd |  |  |  |  |  |  |  |  |  |  | 104 |  |  |  |  |  |  |  |  |  |  |  |  |  |
| Broccoli |  | 190 |  |  |  |  |  |  |  |  |  |  |  |  |  |  |  |  |  |  |  |  |  |  |
| Cabbage |  | 90 |  | 108 | 86 | **0.80** | 160 | 240 | **1.50** |  |  |  | 378 | 28 | **0.07** |  | 49 |  | 124 |  |  |  | 35 |  |
| Carrots | 54 | 47 | **0.86** | 190 |  |  |  |  |  |  |  |  |  |  |  |  |  |  |  |  |  |  |  |  |
| Cauliflower |  | 189 |  |  |  |  |  |  |  |  |  |  |  |  |  |  |  |  |  |  |  |  |  |  |
| Celery |  | 82 |  |  |  |  |  |  |  |  |  |  |  |  |  |  |  |  |  |  |  |  |  |  |
| Chayote | 364 | 40 | **0.11** |  |  |  |  |  |  |  |  |  |  |  |  |  |  |  |  |  |  |  |  |  |
| Chilli, Green |  | 16 |  |  |  |  |  |  |  | 8 | 32 | **4.05** |  |  |  |  |  |  |  |  |  |  |  |  |
| Cilantro | 4 |  |  |  |  |  |  |  |  |  |  |  |  |  |  |  |  |  |  |  |  |  |  |  |
| Cowpea leaves |  |  |  |  |  |  |  |  |  |  |  |  |  |  |  |  |  |  |  |  |  | 42 |  |  |
| Cucumber |  | 178 |  |  |  |  |  |  |  | 235 | 197 | **0.84** |  |  |  |  |  |  |  |  |  |  |  |  |
| Eggplant |  |  |  | 56 | 315 | **5.63** |  | 232 |  | 71 | 129 | **1.83** |  |  |  |  |  |  |  |  |  |  |  |  |
| Entula |  |  |  | 51 | 144 | **2.82** |  | 272 |  |  |  |  |  |  |  |  |  |  |  |  |  |  |  |  |
| Fresh corn |  | 113 |  |  |  |  |  |  |  |  |  |  |  |  |  |  |  |  |  |  |  |  |  |  |
| Garlic |  | 16 |  |  |  |  |  |  |  | 12 | 19 | **1.60** |  |  |  |  |  |  |  |  |  |  |  |  |
| Green beans |  |  |  |  |  |  |  |  |  |  | 115 |  |  |  |  |  |  |  |  |  |  |  |  |  |
| Green maize |  |  |  |  |  |  |  |  |  |  |  |  |  | 154 |  |  |  |  |  | 112 |  |  | 791 |  |
| Hierbas | 80 | 135 | **1.68** |  |  |  |  |  |  |  |  |  |  |  |  |  |  |  |  |  |  |  |  |  |
| Jute leaves |  |  |  |  |  |  |  |  |  |  | 45 |  |  |  |  |  |  |  |  |  |  | 66 |  |  |
| Kale leaves |  |  |  |  |  |  |  |  |  |  |  |  | 399 | 224 | **0.56** | 80 | 79 | **0.99** |  |  |  |  | 378 |  |
| Kachur lati |  |  |  |  |  |  |  |  |  |  | 274 |  |  |  |  |  |  |  |  |  |  |  |  |  |
| Lal Shak |  |  |  |  |  |  |  |  |  | 120 | 130 | **1.08** |  |  |  |  |  |  |  |  |  |  |  |  |
| Lettuce or mixed leaves |  | 39 |  |  |  |  |  |  |  | 87 |  |  |  |  |  |  |  |  |  |  |  |  |  |  |
| Mushrooms |  |  |  |  |  |  |  |  |  |  |  |  |  |  |  |  |  |  |  |  |  |  | 35 |  |
| Okra |  |  |  |  |  |  |  |  |  |  | 108 |  |  |  |  |  |  |  |  |  |  |  |  |  |
| Onion | 112 | 14 | **0.13** | 18 | 35 | **1.94** | 42 | 80 | **1.90** | 70 | 87 | **1.25** | 28 | 49 | **1.75** | 49 | 45 | **0.92** | 28 | 21 | **0.75** | 21 | 259 | **12.3** |
| Onion, leaves | 2 |  |  |  |  |  |  |  |  |  |  |  |  |  |  |  |  |  |  |  |  |  |  |  |
| Pui |  |  |  |  |  |  |  |  |  |  | 72 |  |  |  |  |  |  |  |  |  |  |  |  |  |
| Pumpkin |  |  |  |  |  |  |  | 276 |  |  |  |  |  |  |  |  |  |  |  |  |  |  | 21 |  |
| Pumpkin leaves |  |  |  |  |  |  |  |  |  |  | 86 |  |  |  |  |  |  |  |  |  |  | 75 |  |  |
| Radish leaves | 56 |  |  |  |  |  |  |  |  |  |  |  |  |  |  |  |  |  |  |  |  |  |  |  |
| Ribbed gourd |  |  |  |  |  |  |  |  |  |  | 82 |  |  |  |  |  |  |  |  |  |  |  |  |  |
| Sheem bean |  |  |  |  |  |  |  |  |  | 112 | 165 | **1.48** |  |  |  |  |  |  |  |  |  |  |  |  |
| Spider plant |  |  |  |  |  |  |  |  |  |  |  |  |  |  |  |  |  |  |  |  |  | 13 |  |  |
| Spinach leaves |  |  |  |  |  |  |  |  |  |  | 90 |  |  |  |  | 78 | 35 | **0.45** |  |  |  |  |  |  |
| Squash |  | 176 |  |  |  |  |  |  |  |  |  |  |  |  |  |  |  |  |  |  |  |  | 42 |  |
| Sweet pepper |  |  |  |  |  |  |  | 414 |  |  |  |  |  |  |  |  |  |  |  |  |  |  |  |  |
| Tomato | 63 | 111 | **1.76** | 105 | 112 | **1.07** | 120 | 217 | **1.81** | 154 | 188 | **1.22** | 280 | 133 | **0.48** | 175 | 143 | **0.82** | 77 |  |  | 91 | 294 | **3.23** |
| Water gourd |  |  |  |  |  |  |  |  |  | 147 | 261 | **1.78** |  |  |  |  |  |  |  |  |  |  |  |  |
| **Summary** | | | | | | | | | | | | | | | | | | | | | | | | |
|  | **Western Highlands** | | | **East Uganda** | | | **West Uganda** | | | **Sylhet** | | | **Kitui** | | | **Isiolo** | | | **Marsabit** | | | **Vihiga** | | |
|  | 24HR^6^ | HCES^7^ | All^8^ | 24HR | HCES | All | 24HR | HCES | All | 24HR | HCES | All | 24HR | HCES | All | 24HR | HCES | All | 24HR | HCES |  | 24HR | HCES | All |
| **Number of foods** | 37 | 58 | 68 | 27 | 41 | 46 | 29 | 39 | 46 | 42 | 63 | 76 | 19 | 36 | 41 | 20 | 29 | 34 | 20 | 16 | 24 | 30 | 46 | 55 |
| **% overall agreement^9^** | 39.7 | | | 45.7 | | | 47.8 | | | 38.2 | | | 34.1 | | | 44.1 | | | 50.0 | | | 38.2 | | |
| **Mean ratio of HCES to 24HR maximum portion** | 1.21 | | | 1.63 | | | 1.57 | | | 1.67 | | | 1.29 | | | 0.82 | | | 1.11 | | | 1.61 | | |
| **Median ratio of HCES to 24HR maximum portions** | 1.00 | | | 1.19 | | | 1.39 | | | 1.14 | | | 0.62 | | | 0.78 | | | 0.54 | | | 1.00 | | |

^1^Maximum amount = average serving size x the maximum frequency per week that the food could be selected into any modelled diet

^2^Ratio of the maximum weekly food portion (g/week) from the Household consumption and expenditure (HCES) dataset / the maximum weekly food portion from the 24-hour recall (24HR) datasets for each matched food

^3^Maximum portion sizes (g/week) for each food from the 24HR dataset

^4^Maximum portion sizes (g/week) for each food from the HCES dataset

^5^Ratio = maximum weekly food portion in the HCES dataset / maximum weekly food portion in the 24HR dataset

^6^Number of foods in the food list generated using the 24HR data.

^7^Number of foods in the food list generated using the HCES data.

^8^Total number of foods, including those from both the 24HR and HCES data.

^9^% overall agreement = number of foods that were in both the HCES and 24HR food lists / total number of foods across both food lists x 100

Figure S1: Percent of foods within each food group that was only reported in the HCES-derived food list, the 24-hour recall derived food list and in both datasets across all 8 geographical areas

**Table S2:** Number of matched maximum food portions (g/week)^1^, median ratio of maximum food portions^2^, the percentage of ratios that were within given limits by selected food groups^3^ and the percentage of HCES maximum food portions that were higher than 24-hour maximum food portions^4^

| **Food Groups** | **Number of matched portions** | **Median (25^th^,75^th^) food portion ratio** | **% ratios within 0.9 and 1.10** | **% ratios within 0.75 and 1.25** | **% ratios within 0.5 and 1.50** | **% HCES > 24 HR^4^** |
| --- | --- | --- | --- | --- | --- | --- |
| Added Fat | 12 | 1.09  (0.87, 1.43) | 25.0 | 33.3 | 75.0 | 58.3 |
| Added Sugar | 8 | 0.88  (0.52,1.18) | 12.5 | 25.0 | 75.0 | 72.4 |
| Bakery Products | 5 | 1.42  (1.4, 1.56) | 0 | 0 | 60.0 | 100 |
| Dairy Products | 10 | 0.81  (0.35, 1.03) | 30.0 | 30.0 | 50.0 | 30.0 |
| Fruits | 10 | 0.94  (0.67, 1.29) | 20.0 | 40.0 | 80.0 | 40.0 |
| Grains & grain products | 30 | 0.85  (0.56, 1.18) | 6.7 | 36.7 | 66.7 | 46.7 |
| Legumes, nuts & seeds | 8 | 1.11  (1.0, 1.25) | 37.5 | 62.5 | 100.0 | 62.5 |
| Meat, fish & eggs | 14 | 1.01  (0.5, 1.21) | 14.3 | 35.7 | 50.0 | 57.1 |
| Starchy roots & other plant foods | 17 | 1.28  (0.66, 2.01) | 5.9 | 17.6 | 52.9 | 52.9 |
| Vegetables | 36 | 1.18  (0.81, 1.77) | 13.9 | 38.9 | 50.0 | 59.5 |

^1^number of foods that were in the food lists, for both the paired Household consumption and expenditure (HCES) and 24-hour recall data (24HR) sets for each food group (all regions combined)

^2^ratio of HCES/24HR maximum portion

^3^selected food groups when there were at least 5 matched maximum food portions in the food group across all regions

^4^The percentage of HCES/24HR maximum portion sizes ratios that were greater than 1.0.

**Table S3:** Agreement, in the food groups and food sub-groups modelled, between paired 24HR- and HCES-derived model parameters by geographical area

| **Food Group/ Food sub-group** | **W. Highlands, Guatemala** | | **East Uganda** | | **West Uganda** | | **Sylhet, Bangladesh** | | **Kitui, Kenya** | | **Isiolo, Kenya** | | **Marsabit, Kenya** | | **Vihiga, Kenya** | | **All** | |
| --- | --- | --- | --- | --- | --- | --- | --- | --- | --- | --- | --- | --- | --- | --- | --- | --- | --- | --- |
|  | 24HR^1^ | HCES^2^ | 24HR | HCES | 24HR | HCES | 24HR | HCES | 24HR | HCES | 24HR | HCES | 24HR | HCES | 24HR | HCES | % agree^3^ | |
| **Added Fats** | 1 | 1 | 1 | 1 | 1 | 1 | 1 | 1 | 1 | 1 | 1 | 1 | 1 | 1 | 1 | 1 | **100** | |
| Butter, ghee, margarine, unfortified |  | 1 |  |  |  | 1 |  |  |  | 1 | 1 | 1 | 1 | 1 | 1 | 1 | 50.0 | |
| Margarine, fortified |  |  |  | 1 |  | 1 |  |  | 1 |  | 1 |  | 1 |  | 1 | 1 | 16.7 | |
| Vegetable oil (fortified or unfortified) | 1 | 1 | 1 | 1 | 1 | 1 | 1 | 1 | 1 | 1 | 1 | 1 | 1 | 1 | 1 | 1 | 100 | |
| **Added sugars** | 1 | 1 | 1 | 1 | 1 | 1 | 1 | 1 | 1 | 1 | 1 | 1 | 1 | 1 | 1 | 1 | **100** | |
| Honey, syrup |  |  |  |  |  |  |  |  |  | 1 |  |  |  |  |  |  | 0 | |
| Sugar | 1 | 1 | 1 | 1 | 1 | 1 | 1 | 1 | 1 | 1 | 1 | 1 | 1 | 1 | 1 | 1 | 100 | |
| **Bakery Products** | 1 | 1 | 1 | 1 | 1 | 1 | 1 |  |  | 1 |  | 1 |  |  | 1 | 1 | **57.1** | |
| Pancakes, waffles, crackers |  |  |  |  |  |  | 1 |  |  |  |  |  |  |  |  |  | 0 | |
| Refined grain bread | 1 | 1 | 1 | 1 | 1 | 1 |  |  |  | 1 |  | 1 |  |  | 1 | 1 | 66.7 | |
| Sweetened bakery products |  |  | 1 |  |  |  | 1 |  |  |  |  | 1 |  |  | 1 | 1 | 25.0 | |
| **Beverages (non- or blended dairy)** | 1 | 1 |  | 1 |  | 1 |  | 1 |  | 1 |  |  |  | 1 |  | 1 | **14.3** | |
| Chocolate beverage or powder mix |  |  |  |  |  |  |  |  |  |  |  |  |  |  |  | 1 | 0 | |
| Juices - commercial |  | 1 |  |  |  |  |  | 1 |  |  |  |  |  |  |  |  | 0 | |
| Other beverages |  |  |  |  |  |  |  |  |  |  |  |  |  | 1 |  |  | 0 | |
| Sugar-sweetened beverages | 1 | 1 |  | 1 |  | 1 |  |  |  | 1 |  |  |  |  |  | 1 | 20.0 | |
| **Composites (mixed food groups)** | 1 | 1 | 1 |  | 1 |  | 1 | 1 | 1 |  |  |  |  |  | 1 |  | **33.3** | |
| Broths or soups | 1 | 1 | 1 |  | 1 |  |  |  | 1 |  |  |  |  |  | 1 |  | 20.0 | |
| Other composites |  |  |  |  | 1 |  | 1 | 1 |  |  |  |  |  |  |  |  | 50.0 | |
| **Dairy products** | 1 | 1 | 1 | 1 | 1 | 1 | 1 | 1 | 1 | 1 | 1 | 1 | 1 | 1 | 1 | 1 | **100** | |
| Cheese | 1 | 1 |  |  |  |  |  |  |  |  |  |  |  |  |  |  | 100 | |
| Cream, sour cream |  | 1 |  |  |  |  |  |  |  |  |  |  |  |  |  |  | 0 | |
| Milk, fluid or powdered | 1 | 1 | 1 | 1 | 1 | 1 | 1 | 1 | 1 | 1 | 1 | 1 | 1 | 1 | 1 | 1 | 100 | |
| **Fruits** | 1 | 1 | 1 | 1 | 1 | 1 | 1 | 1 |  | 1 | 1 | 1 |  |  | 1 | 1 | **85.7** | |
| Other fruit | 1 | 1 | 1 | 1 | 1 | 1 | 1 | 1 |  | 1 | 1 | 1 |  |  | 1 | 1 | 85.7 | |
| Vitamin A source fruit |  |  |  | 1 |  | 1 |  | 1 |  | 1 |  | 1 |  |  |  | 1 | 0 | |
| Vitamin C-rich fruit | 1 | 1 |  | 1 |  | 1 | 1 | 1 |  | 1 |  |  |  |  | 1 | 1 | 50.0 | |
| **Grains & grain products** | 1 | 1 | 1 | 1 | 1 | 1 | 1 | 1 | 1 | 1 | 1 | 1 | 1 | 1 | 1 | 1 | **100** | |
| Fortified grains and products | 1 | 1 |  |  |  |  |  |  |  |  |  |  | 1 |  |  |  | 50.0 | |
| Refined grains & products, unfortified | 1 | 1 | 1 | 1 | 1 | 1 | 1 | 1 | 1 | 1 | 1 | 1 | 1 | 1 | 1 | 1 | 100 | |
| Whole grains and products, unenriched/unfortified | 1 | 1 | 1 | 1 | 1 | 1 | 1 | 1 | 1 | 1 | 1 | 1 | 1 | 1 | 1 | 1 | 100 | |
| **Human Milk** | 1 | 1 | 1 | 1 | 1 | 1 | 1 | 1 | 1 | 1 | 1 | 1 | 1 | 1 | 1 | 1 | **100** | |
| Breastmilk | 1 | 1 | 1 | 1 | 1 | 1 | 1 | 1 | 1 | 1 | 1 | 1 | 1 | 1 | 1 | 1 | 100 | |
| **Legumes, nuts & seeds** | 1 | 1 | 1 | 1 | 1 | 1 | 1 | 1 | 1 | 1 | 1 | 1 | 1 | 1 | 1 | 1 | **100** | |
| Cooked beans, lentils, peas | 1 | 1 | 1 | 1 | 1 | 1 | 1 | 1 | 1 | 1 | 1 | 1 | 1 | 1 | 1 | 1 | 100 | |
| Nuts, seeds & unsweetened products |  |  | 1 | 1 | 1 | 1 |  | 1 |  |  |  |  |  |  |  | 1 | 50 | |
| Soybeans and products |  |  |  |  | 1 |  |  |  |  |  |  |  |  |  |  |  | 0 | |
| **Food Group/ Food sub-group** | **W. Highlands** | | **East Uganda** | | **West Uganda** | | **Sylhet** | | **Kitui** | | **Isiolo** | | **Marsabit** | | **Vihiga** | | **All** | |
|  | 24HR | HCES | 24HR | HCES | 24HR | HCES | 24HR | HCES | 24HR | HCES | 24HR | HCES | 24HR | HCES | 24HR | HCES | % Agree | |
| **Meat, fish & Eggs** | 1 | 1 | 1 | 1 | 1 | 1 | 1 | 1 |  | 1 | 1 | 1 | 1 | 1 | 1 | 1 | **87.5** | |
| Eggs | 1 | 1 |  | 1 | 1 | 1 | 1 | 1 |  | 1 | 1 |  |  |  |  | 1 | 42.9 | |
| Fish without bones |  |  |  | 1 |  | 1 | 1 | 1 |  |  |  |  |  |  |  | 1 | 25.0 | |
| Organ meat |  | 1 |  |  |  |  |  |  |  |  |  | 1 |  |  |  | 1 | 0 | |
| Pork |  | 1 |  | 1 |  | 1 |  |  |  |  |  |  |  |  |  |  | 0 | |
| Poultry, rabbit | 1 | 1 |  | 1 |  |  |  | 1 |  |  |  |  |  |  |  | 1 | 25.0 | |
| Processed meat |  | 1 |  |  |  |  |  |  |  |  |  |  |  |  |  |  | 0 | |
| Red meat |  | 1 |  | 1 | 1 | 1 |  | 1 |  | 1 |  | 1 | 1 | 1 | 1 | 1 | 37.5 | |
| Seafood |  |  |  |  |  |  | 1 |  |  |  |  |  |  |  |  |  | 0 | |
| Small, whole fish, w/bones |  |  | 1 | 1 |  | 1 | 1 | 1 |  |  |  |  |  |  | 1 | 1 | 75.0 | |
| **Miscellaneous** |  | 1 |  |  |  |  |  |  |  |  |  |  |  |  |  |  | **0** | |
| Savory spreads, sauces, pastes |  | 1 |  |  |  |  |  |  |  |  |  |  |  |  |  |  | 0 | |
| **Starchy roots & other starchy plant foods** | 1 | 1 | 1 | 1 | 1 | 1 | 1 | 1 | 1 | 1 | 1 | 1 | 1 | 1 | 1 | 1 | **100** | |
| Other starchy plant foods | 1 | 1 | 1 | 1 | 1 | 1 | 1 | 1 | 1 | 1 | 1 | 1 | 1 | 1 | 1 | 1 | 100 | |
| **Sweetened snacks & desserts** |  | 1 |  |  |  |  | 1 |  |  | 1 |  | 1 |  |  |  | 1 | **0** | |
| Sweet snack foods (candy and chocolate) |  | 1 |  |  |  |  | 1 |  |  | 1 |  | 1 |  |  |  | 1 | 0 | |
| **Vegetables** | 1 | 1 | 1 | 1 | 1 | 1 | 1 | 1 | 1 | 1 | 1 | 1 | 1 | 1 | 1 | 1 | 100 | |
| Condiment vegetables | 1 | 1 |  |  |  |  | 1 | 1 |  |  |  |  |  |  |  |  | 100 | |
| Other vegetables | 1 | 1 | 1 | 1 | 1 | 1 | 1 | 1 | 1 | 1 | 1 | 1 | 1 | 1 | 1 | 1 | 100 | |
| Vitamin A source dark green leafy vegetables | 1 | 1 | 1 | 1 | 1 | 1 | 1 | 1 | 1 | 1 | 1 | 1 |  |  | 1 | 1 | 100 | |
| Vitamin A source other vegetables | 1 | 1 |  |  |  | 1 |  |  |  |  |  |  |  |  | 1 | 1 | 66.7 | |
| Vitamin C-rich vegetables | 1 | 1 | 1 | 1 | 1 | 1 | 1 | 1 | 1 | 1 | 1 | 1 | 1 |  | 1 | 1 | 87.5 | |
| **Summary** | | | | | | | | | | | | | | | | | **Mean^3^**  **^24HR HCES^** | |
| Number of food groups | 13 | 15 | 12 | 12 | 12 | 12 | 13 | 12 | 9 | 13 | 10 | 12 | 9 | 10 | 12 | 13 | 11 | 12 |
| Number of food sub-groups | 22 | 31 | 17 | 24 | 19 | 25 | 22 | 23 | 13 | 21 | 15 | 19 | 14 | 12 | 21 | 29 | 18 | 23 |
| % overall agreement^4^ food groups | 86.7 | | 84.6 | | 84.6 | | 78.6 | | 57.1 | | 83.3 | | 90.0 | | 78.6 | | 80.4 | |
| % overall agreement food sub-groups | 71.0 | | 57.7 | | 57.1 | | 66.7 | | 47.8 | | 61.9 | | 73.3 | | 66.7 | | 62.8 | |

^1^24HR -the number 1 indicates that the food group or food sub-group was included as a model parameter in the analyses done using the 24-hour recall data.

^2^HCES -the number 1 indicates that the food group or food sub-group was included as a model parameter in the analyses done using the Household consumption and expenditure (HCES) data.

^3^Mean across all geographical areas

^4^% overall agreement = number that were include as both 24HR and HCES model parameters / total number included as either HCES or 24HR model parameters x 100

**Table S4:** Summary of agreement^1^ between paired 24HR and HCES derived model parameters at the Food Group level

| **Food Group** | **Agreement^1^** | **Only in 24HR** | **Only in HCES** | **Not modelled** |
| --- | --- | --- | --- | --- |
| **Added fats** | All data pairs |  |  |  |
| **Added sugars** | 6 data pairs |  | Kitui, Marsabit |  |
| **Bakery & breakfast cereals** | 4 data pairs | Sylhet | Isiolo, Kitui | Marsabit |
| **Beverages (non-dairy or blended dairy)** | 1 data pair |  | Eastern and Western Uganda, Vihiga, Kitui, Marsabit and Sylhet | Isiolo |
| **Composites (mixed food groups)** | 1 data pair | Eastern and Western Uganda, Kitui, Marsabit, Vihiga |  | Isiolo, Sylhet |
| **Dairy products** | All data pairs |  |  |  |
| **Fruits** | 6 data pairs |  | Kitui | Marsabit |
| **Grains & grain products** | All data pairs |  |  |  |
| **Human milk** | All data pairs |  |  |  |
| **Legumes, nuts & seeds** | All data pairs |  |  |  |
| **Meat, fish & eggs** | 7 data pairs |  | Kitui |  |
| **Miscellaneous** |  |  | Western Highlands | 7 data pairs |
| **Other composites** | Sylhet |  |  | 7 data pairs |
| **Starchy roots & plant foods** | All data pairs |  |  |  |
| **Sweetened snacks & desserts** |  | Sylhet | Isiolo, Kitui, Vihiga, Western Highlands | East Uganda, Marsabit, West Uganda |
| **Vegetables** | All data pairs |  |  |  |

^1^Both the HCES and 24 HR derived model input parameters included the food group.

Table S5: Number of modelled nutrients for which each food sub-group was identified as a good nutrient source^1^ and the percentage eligible and overall agreement between 24HR^2^ and HCES^3^ food list pairs by geographical area.

| **Food Sub-groups** | **Western Highlands, Guatemala** | | **Eastern Uganda** | | **Western Uganda** | | **Sylhet, Bangladesh** | | **Kitui, Kenya** | | **Isiolo, Kenya** | | **Marsabit, Kenya** | | **Vihiga, Kenya** | | **% overall agreement^4^** |
| --- | --- | --- | --- | --- | --- | --- | --- | --- | --- | --- | --- | --- | --- | --- | --- | --- | --- |
|  | 24HR | HCES | 24HR | HCES | 24HR | HCES | 24HR | HCES | 24HR | HCES | 24HR | HCES | 24HR | HCES | 24HR | HCES |  |
| Butter, ghee, margarine (unfortified) | . | 0 | . | . | 0 | 0 | . | . | . | 0 | 0 | 0 | 0 | 0 | 0 | 0 | 100 |
| Margarine (fortified) | . | . | . | 0 | 0 | 0 | . | . | 2 | . | 0 | . | 0 | . | 0 | 0 | 83.3 |
| Vegetable oil (fortified) | . | . | 0 | 0 | 0 | 0 | . | . | 0 | 0 | 0 | 0 | 0 | 1 | 0 | 0 | 83.3 |
| Vegetable oil (unfortified) | 0 | 0 | . | . |  |  | 0 | 0 | . | . | . | . | . | . | . | . | 100 |
| Sugar | 0 | 0 | 0 | 0 | 0 | 0 | 0 | 0 | 0 | 0 | 0 | 0 | 0 | 0 | 0 | 0 | 100 |
| Refined grain bread | 0 | 5 | 6 | 8 | 6 | 6 | . | . | . | 0 | . | 0 | . | . | 0 | 1 | 66.7 |
| Sweet bakery products | . | . | 0 | . | . | . | 0 | . | . | . | . | 0 | . | . | 0 | 0 | 100 |
| Sugar-sweetened drinks | 0 | 0 | . | 0 | 0 | 0 | . | . | . | 0 | . | . | . | . | . | 0 | 100 |
| Broths or soups | 0 | 2 | 0 | . | . | . | . | . | 4 | . | . | . | . | . | 0 | . | 50 |
| Fluid or powdered milk | 0 | 0 | 6 | 4 | 5 | 5 | 4 | 6 | 5 | 3 | 10 | 2 | 10 | 2 | 4 | 2 | 100 |
| Other fruit | 0 | 0 | 0 | 0 | 1 | 1 | 0 | 0 | . | 0 | 0 | 0 | . | . | 0 | 0 | 100 |
| Vitamin A source fruit | . | 0 | . | 0 | 0 | 0 | . | 0 | . | 10 | . | 0 | . | . | . | 2 | 71.4 |
| Vitamin C-rich fruit | 0 | 0 | 0 | 1 | 0 | 0 | 1 | 1 | . | 0 | . | . | . | . | 0 | 1 | 66.7 |
| Enriched/fortified grains & products | 11 | 6 | . | . | . | . | . | . | . | . | . | . | 4 | . | . | . | 50.0 |
| Refined grains and products (unfortified) | 8 | 8 | 0 | 0 | 0 | 0 | . | . | 5 | 0 | 0 | 0 | 0 | 0 | 0 | 0 | 85.7 |
| Whole grains and products (unfortified) | 3 | 8 | 5 | 7 | 5 | 5 | 8 | 7 | 7 | 7 | 5 | 7 | 2 | 7 | 7 | 7 | 100 |
| Breastmilk | 10 | 10 | 10 | 10 | 10 | 10 | 10 | 10 | 10 | 10 | 10 | 10 | 10 | 10 | 10 | 10 | 100 |
| Beans, lentils, peas | 2 | 4 | 3 | 3 | 7 | 7 | 3 | 2 | 6 | 7 | 8 | 8 | 8 | 6 | 4 | 7 | 100 |
| Nuts, seeds, not sweet | . | . | 5 | 0 | 0 | 0 | . | 0 | . | . | . | . | . | . | . | 1 | 50.0 |
| Eggs | 2 | 2 | . | 8 | 0 | 0 | 1 | 0 | . | 0 | 0 | . | . | . | . | 0 | 71.4 |
| Fish without bones | . | . | . | 0 | 0 | 0 | 0 | 2 | . | . | . | . | . | . | . | 0 | 75.0 |
| Organ meat | . | 4 | . | . | . | . | . | . | . |  | . | 7 | . | . | . | 5 | 0 |
| Pork | . | 0 | . | 0 | 0 | 0 | . | . | . | . | . | . | . | . | . | . | 100 |
| Poultry, rabbit | 0 | 0 | . | 0 | . | . | . | . | . | . | . | . | . | . | . | 0 | 100 |
| Red meat | . | 0 | . | 2 | 7 | 7 | . | . | . | 1 | . | 3 | 5 | 8 | 2 | 2 | 57.1 |
| Small, whole fish | . | . | 6 | 7 | 0 | 0 | 3 | 2 | . | . | . | . | . | . | 7 | 2 | 100 |
| Other composites | . | . | . | . | . | . | 0 | 0 | . | . | . | . | . | . | . | . | 100 |
| Other starchy plant foods | 0 | 0 | 8 | 0 | 5 | 5 | 6 | 0 | 3 | 2 | 5 | 2 | 5 | 2 | 3 | 5 | 75.0 |
| Condiment vegetables | 0 | 0 | 0 | 0 | 0 | 0 | 0 | 1 | . | . | . | . | . | . | . | 0 | 80.0 |
| Other vegetables | 0 | 3 | 0 | 0 | 1 | 4 | 7 | 6 | 1 | 3 | 3 | 1 | 0 | 2 | 4 | 2 | 75.0 |
| Vitamin A source dark green leafy vegetables | 5 | 5 | 8 | 3 | 7 | 6 | 7 | 9 | 10 | 8 | 7 | 5 | . | . | 9 | 10 | 100 |
| Vitamin A source other vegetables | 0 | 0 | 0 | 2 | 0 | 0 | . | . | . | . | . | . | . | . | 1 | 0 | 50.0 |
| Vitamin C-rich vegetables | 0 | 0 | 1 | 2 | 7 | 7 | 0 | 7 | 6 | 0 | . | 1 | 2 | . | 0 | 0 | 50.0 |
| No. FSGs^5^ identified as good nutrient sources | 7 | 11 | 10 | 12 | 11 | 11 | 10 | 11 | 11 | 9 | 7 | 10 | 8 | 8 | 10 | 14 |  |
| No. of eligible FSG pairs | 21 | | 18 | | 26 | | 17 | | 11 | | 12 | | 11 | | 21 | |  |
| No. of overall FSG pairs | 26 | | 28 | | 26 | | 20 | | 22 | | 20 | | 14 | | 30 | |  |
| No. eligible FSG’s agreed | 18 | | 14 | | 26 | | 12 | | 9 | | 12 | | 9 | | 18 | |  |
| No. overall FSG’s agreed | 22 | | 22 | | 26 | | 15 | | 16 | | 17 | | 10 | | 24 | | **Mean** |
| Eligible Agreement^6^, % | 85.7 | | 77.8 | | 100 | | 70.6 | | 81.8 | | 100 | | 81.8 | | 85.7 | | 85.4 |
| Overall Agreement, % | 84.6 | | 78.6 | | 100 | | 75.0 | | 72.7 | | 85.0 | | 71.4 | | 80.0 | | 80.9 |

^1^Food sub-groups were defined as a ‘good source’ of a modelled nutrient if they provided ≥5% of that nutrient in the module 2, nutritionally best diet.

^2^24HR – the analyses were done using model parameters generated from individual 24-hour recall dietary dataset.

^3^HCES - the analyses were done using model parameters generated from household consumption and expenditure dietary dataset.

^4^% Overall agreement - data pairs ‘agreed’ if food sub-groups were a good source of at least one modelled nutrient across both dataset pairs or it was not a good source of any modelled nutrients for either dataset pair. Percentage agreement was calculated as the number of food sub-groups for which there was agreement across both dataset pairs, divided by the number of food sub-groups (i.e., food sub-groups which were present in at least one of the paired datasets).

^5^FSG – food sub-group

^6^% Eligible agreement - data pairs ‘agreed’ if food sub-groups were a good source of at least one modelled nutrient across both dataset pairs or it was not a good source of any modelled nutrients for either dataset pair. Percentage agreement was calculated as the number of food sub-groups for which there was agreement across both dataset pairs, divided by the number of eligible food sub-group pairs (i.e., food sub-groups which were present in both paired datasets).

**Table S6:** Draft individual food-based recommendations (expressed as the number of average portions per food sub-group per week) tested (Module 3, minimized analyses) by geographic area and data source.

| **Food Group** | **Food Sub-Group** | **W. Highlands, Guatemala** | | **East Uganda** | | **West Uganda** | | **Sylhet, Bangladesh** | | **Kitui, Kenya** | | **Isiolo, Kenya** | | **Marsabit, Kenya** | | **Vihiga, Kenya** | |
| --- | --- | --- | --- | --- | --- | --- | --- | --- | --- | --- | --- | --- | --- | --- | --- | --- | --- |
|  |  | **24HR** | **HCES** | **24HR** | **HCES** | **24HR** | **HCES** | **24HR** | **HCES** | **24HR** | **HCES** | **24HR** | **HCES** | **24HR** | **HCES** | **24HR** | **HCES** |
| Added fats | Vegetable oil (fortified) |  |  |  |  |  |  |  |  |  |  |  |  |  | 7 |  |  |
| Bakery & cereals | Refined grain bread, unfortified |  | 7 | 2 | 10 | 4 | 5 |  |  |  |  |  |  |  |  |  |  |
| Composites (mixed) | Broths |  |  |  |  |  |  |  |  |  |  | 7 |  |  |  |  |  |
| Dairy | Fluid/powdered milk, unfortified |  |  | 7 | 5 | 7 | 6 | 4 | 5 | 7 | 7 | 7 | 14 | 14 | 7 | 7 | 14 |
| Fruits | Vitamin A source fruit |  |  |  |  |  |  |  |  |  |  |  | 7 |  |  |  |  |
|  | Vitamin C-rich fruit |  |  |  |  |  |  | 3 | 8 |  |  |  |  |  |  |  |  |
| Grains | Enriched/fortified grains | 7 | 2 |  |  |  |  |  |  |  |  |  |  | 3 |  |  |  |
|  | Refined grains, unfortified | 14 | 2 |  |  |  |  |  |  |  |  | 7 |  |  |  |  |  |
|  | Whole grains, unfortified | 21 | 21 | 14 | 14 | 14 | 10 | 14 | 14 | 7 | 14 | 14 | 21 | 7 | 14 | 12 | 28 |
| Legumes, nuts & seeds | Cooked beans, lentils, peas | 7 | 7 | 5 | 6 | 7 | 6 | 6 | 6 | 7 | 14 | 5 | 14 | 7 | 7 | 3 | 14 |
|  | Nuts, seeds, unsweetened |  |  | 5 |  |  |  |  |  |  |  |  |  |  |  |  |  |
| Meat, fish & eggs | Eggs | 6 | 3 |  | 5 |  |  |  |  |  |  |  |  |  |  |  |  |
|  | Organ meat |  | 1 |  |  |  |  |  |  |  | 7 |  |  |  |  |  | 7 |
|  | Red meat |  |  |  | 1 | 1 | 2 |  |  |  | 7 |  | 7 | 2 | 7 | 2 | 7 |
|  | Small, whole fish, with bones |  |  | 7 | 5 |  |  | 7 | 5 |  |  |  |  |  |  | 4 | 7 |
| Starchy roots & plants | Other starchy plant foods |  |  | 7 |  | 7 | 7 | 21 |  | 7 | 14 | 4 | 14 | 7 | 7 | 7 | 21 |
| Vegetables | Other vegetables |  |  |  |  | 7 | 7 | 10 | 7 | 14 |  |  | 21 | 7 | 14 | 14 |  |
|  | Vit A source green leafy veg | 7 | 3 | 7 | 4 | 14 | 7 | 6 | 5 | 7 | 14 | 7 | 7 |  |  | 7 | 7 |
|  | Vitamin C-rich vegetables |  |  |  |  |  |  |  | 7 |  | 7 | 3 |  | 4 |  |  |  |
| **Number of Food Based Recommendations tested** | | **6** | **8** | **8** | **8** | **8** | **8** | **8** | **8** | **6** | **8** | **8** | **8** | **8** | **7** | **8** | **8** |

**Table S7:** Final food-based recommendations (FBRs) selected (expressed as grams per week) by geographical area

| **Recommended food sub-group** | **W. Highlands, Guatemala**  g/w | | **East Uganda**  g/w | | **West Uganda**  g/w | | **Sylhet, Bangladesh**  g/w | | **Kitui, Kenya**  g/w | | **Isiolo, Kenya**  g/w | | **Marsabit, Kenya**  g/w | | **Vihiga, Kenya**  g/w | |
| --- | --- | --- | --- | --- | --- | --- | --- | --- | --- | --- | --- | --- | --- | --- | --- | --- |
|  | **24HR** | **HCES** | **24HR** | **HCES** | **24HR** | **HCES** | **24HR** | **HCES** | **24HR** | **HCES** | **24HR** | **HCES** | **24HR** | **HCES** | **24HR** | **HCES** |
| Refined grain bread, unfortified |  |  | 92 | 400 | 110 | 205 |  |  |  |  |  |  |  |  |  |  |
| Fluid/powdered milk, unfortified |  |  | 546 | 400 | 560 | 504 | 224 | 315 | 567 | 434 | 560 | 210 | 700 | 490 | 406 | 700 |
| Enriched/fortified grains | 77 | 42 |  |  |  |  |  |  |  |  |  |  |  |  |  |  |
| Refined grains, unfortified |  | 94 |  |  |  |  |  |  |  |  |  |  |  |  |  |  |
| Whole grains, unfortified | 441 | 420 | 1988 | 1442 | 1372 | 1070 |  |  | 524 | 714 | 315 | 560 | 315 | 406 | 336 | 448 |
| Cooked beans, lentils, peas |  |  | 210 | 312 | 252 | 246 | 66 | 90 | 105 | 252 | 287 | 420 | 231 | 175 | 81 | 77 |
| Eggs | 108 | 108 |  |  |  |  |  |  |  |  |  |  |  |  |  |  |
| Organ meat |  |  |  |  |  |  |  |  |  |  |  | 35 |  |  |  |  |
| Red meat |  |  |  |  | 30 | 100 |  |  |  |  |  |  | 80 | 140 |  |  |
| Small, whole fish, with bones |  |  | 84 | 85 |  |  | 24 | 25 |  |  |  |  |  |  | 80 | 70 |
| Other starchy plant foods |  |  |  |  |  |  |  |  |  |  |  |  |  |  | 588 | 560 |
| Other vegetables |  |  |  |  |  |  |  |  |  | 420 |  |  |  | 140 |  |  |
| Vit A source dark green leafy veg | 119 | 135 | 140 | 80 | 112 | 147 |  |  | 385 | 329 | 161 | 280 |  |  | 350 | 378 |
| Vitamin C-rich vegetables |  |  |  |  |  |  |  |  | 378 |  |  |  | 124 |  |  |  |
| **Summary** | | | | | | | | | | | | | | | | |
| Number of FBRs^1^ | 4 | 5 | 6 | 6 | 6 | 6 | 3 | 3 | 5 | 5 | 4 | 5 | 5 | 5 | 6 | 6 |
| Total number of FBRs^2^ | 5 | | 6 | | 6 | | 3 | | 6 | | 5 | | 6 | | 6 | |
| Total number of eligible FBRs^3^ | 4 | | 6 | | 6 | | 3 | | 4 | | 4 | | 5 | | 6 | |
| Number matched FBRs^4^ | 4 | | 6 | | 6 | | 3 | | 4 | | 4 | | 4 | | 6 | |
| Eligible agreement^5^, % | 100 | | 100 | | 100 | | 100 | | 100 | | 100 | | 80 | | 100 | |
| Overall agreement^6^, % | 80 | | 100 | | 100 | | 100 | | 67 | | 80 | | 67 | | 100 | |

^1^ number of FBRs were the number of individual food-based recommendations selected in each final set of FBRs by dataset type and geographical area.

^2^ total number of FBRs are the number of individual food-based recommendations selected, for testing (Module 3 minimized combined analyses) in at least one of the data set pairs by geographical area.

^3^ eligible FBRs are the total number of FBRs selected for testing (Module 3 minimized combined analyses) across both dataset pairs by geographical area (i.e., it does not count FBRs that were not tested in both dataset pair analyses).

^4^ the number of individual FBRs included in both paired sets of FBRs.

^5^ % eligible agreement = the number of matched individual FBRs / the total number of eligible individual FBRs, where eligible individual FBRs were those selected for testing in both paired analyses.

^6^ % overall agreement = the number of matched FBRs / total number of individual FBRs x 100; where the total number of individual FBRs were those selected for testing in at least one of the data set pairs.

**Table S8:** Nutrients that remained below 65% of the recommended levels when the final sets of food-based recommendations were tested (module 3, minimized nutrient values) and data pair percent agreements.

| **Nutrients** | **W. Highlands, Guatemala**  g/w | | | | **East Uganda**  g/w | | | | **West Uganda**  g/w | | | | **Sylhet, Bangladesh**  g/w | | | | **Kitui, Kenya**  g/w | | | | **Isiolo, Kenya**  g/w | | | | **Marsabit, Kenya**  g/w | | | | **Vihiga, Kenya**  g/w | | | **Overall agreement**  **%** |
| --- | --- | --- | --- | --- | --- | --- | --- | --- | --- | --- | --- | --- | --- | --- | --- | --- | --- | --- | --- | --- | --- | --- | --- | --- | --- | --- | --- | --- | --- | --- | --- | --- |
|  | **24HR^1^** | | **HCES^2^** | | **24HR** | | **HCES** | | **24HR** | | **HCES** | | **24HR** | | **HCES** | | **24HR** | | **HCES** | | **24HR** | | **HCES** | | **24HR** | | **HCES** | | **24HR** | | **HCES** |  |
| Calcium |  | |  | |  | |  | |  | |  | | 1 | | 1 | |  | | 1 | |  | | 1 | |  | | 1 | | 1 | | 1 | 62.5 |
| Thiamin |  | |  | |  | |  | |  | |  | | 1 | | 1 | |  | |  | |  | |  | |  | |  | |  | |  | 100 |
| Niacin |  | |  | |  | |  | |  | |  | | 1 | | 1 | | 1 | | 1 | | 1 | | 1 | | 1 | | 1 | |  | | 1 | 87.5 |
| Vitamin B6 |  | |  | |  | |  | |  | |  | | 1 | | 1 | |  | |  | |  | |  | |  | |  | |  | |  | 100 |
| Folate |  | |  | | 1 | |  | |  | |  | | 1 | | 1 | |  | |  | |  | |  | |  | |  | |  | |  | 87.5 |
| Vitamin B12 |  | |  | |  | |  | |  | |  | | 1 | |  | | 1 | | 1 | |  | |  | |  | |  | |  | |  | 87.5 |
| Zinc |  | |  | | 1 | | 1 | | 1 | | 1 | | 1 | | 1 | | 1 | | 1 | | 1 | | 1 | | 1 | | 1 | | 1 | | 1 | 100 |
| Iron | 1 | | 1 | | 1 | | 1 | | 1 | | 1 | | 1 | | 1 | | 1 | | 1 | | 1 | | 1 | | 1 | | 1 | | 1 | | 1 | 100 |
| **Summary** | | | | | | | | | | | | | | | | | | | | | | | | | | | | | | | | **Mean** |
| Number of “problem nutrients” | 1 | 1 | | 3 | | 2 | | 2 | | 2 | | 8 | | 7 | | 4 | | 5 | | 3 | | 4 | | 3 | | 4 | | 3 | | 4 | | 3.5 |
| Total number of problem nutrients across dataset pairs | 1 | | | 3 | | | | 2 | | | | 8 | | | | 5 | | | | 4 | | | | 4 | | | | 4 | | | | 3.9 |
| Number of matched problem nutrients | 1 | | | 2 | | | | 2 | | | | 7 | | | | 4 | | | | 3 | | | | 3 | | | | 3 | | | | 3.1 |
| Eligible agreement^3^, % | 100 | | | 66.7 | | | | 100 | | | | 87.5 | | | | 80.0 | | | | 75.0 | | | | 75.0 | | | | 75.0 | | | | 82.5 |
| Overall agreement^4^, % | 100 | | | 91.7 | | | | 100 | | | | 91.7 | | | | 91.7 | | | | 91.7 | | | | 91.7 | | | | 91.7 | | | | 93.8 |

^1^value of 1 indicates the nutrient remained below 65% of its recommended level (“inadequate nutrient”), when the final set of food-based recommendations were tested (minimized Module 3), using the 24-hour recall data defined model parameters.

^2^value of 1 indicates the nutrient remained below 65% of the recommended levels, when the final set of food-based recommendations were tested (minimized Module 3), using the household consumption and expenditure survey data defined model parameters.

^3^Eligble percent agreement = number of matched “inadequate nutrients” / total number of “inadequate nutrients” identified across the paired HCES and 24HR analyses x 100; where “inadequate nutrients” were those <65% of their recommended intake levels when tested (minimized Module 3 analyses).

^4^Overall percent agreement = number of correctly categorized nutrients / total number of nutrients modelled x 100; where the category was “inadequate” (yes or no) and the denominator was the total number of nutrients modelled (n=12 nutrients).
